# P450s controlling metabolic bifurcations in plant terpene specialized metabolism

**DOI:** 10.1007/s11101-017-9530-4

**Published:** 2017-09-12

**Authors:** Aparajita Banerjee, Björn Hamberger

**Affiliations:** Department of Biochemistry and Molecular Biology, Michigan State University, 603 Wilson Road, East Lansing, MI 48824 USA

**Keywords:** Terpenoid specialized metabolites, Pathway bifurcation, Regio-specificity, Stereo-specificity, Promiscuity, Orthologs, Biosynthetic, Metabolic diversity, Synthetic Biology, Metabolic engineering, Biotechnology

## Abstract

**Abstract:**

Catalyzing stereo- and regio-specific oxidation of inert hydrocarbon backbones, and a range of more exotic reactions inherently difficult in formal chemical synthesis, cytochromes P450 (P450s) offer outstanding potential for biotechnological engineering. Plants and their dazzling diversity of specialized metabolites have emerged as rich repository for functional P450s with the advances of deep transcriptomics and genome wide discovery. P450s are of outstanding interest for understanding chemical diversification throughout evolution, for gaining mechanistic insights through the study of their structure–function relationship, and for exploitation in Synthetic Biology. In this review, we highlight recent developments and examples in the discovery of plant P450s involved in the biosynthesis of industrially relevant monoterpenoids, sesquiterpenoids, diterpenoids and triterpenoids, throughout 2016 and early 2017. Examples were selected to illustrate the spectrum of value from commodity chemicals, flavor and fragrance compounds to pharmacologically active terpenoids. We focus on a recently emerging theme, where P450s control metabolic bifurcations and chemical diversity of the final product profile, either within a pathway, or through neo-functionalization in related species. The implications may inform approaches for rational assembly of recombinant pathways, biotechnological production of high value terpenoids and generation of novel chemical entities.

**Graphical Abstract:**

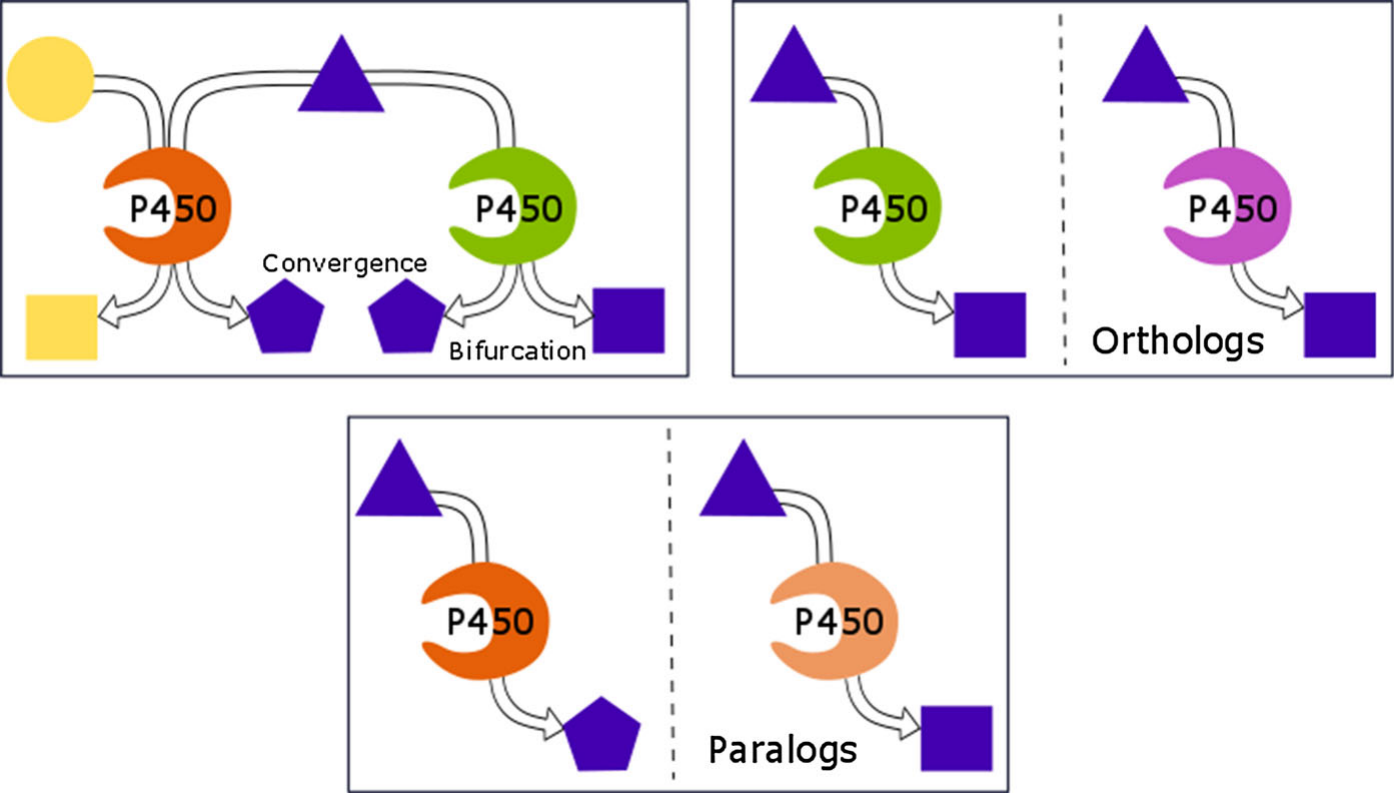

## Introduction

The few plant pathways to specialized metabolites found in text books appear as discrete cascades to end-point molecules of interest (e.g. morphine biosynthesis). A paradigm of linear pathways still appears applicable to the universally shared pathways providing the C_5_ building blocks for terpenoid metabolism across all life kingdoms. However, plant pathways of the specialized metabolism have impressively diversified throughout evolution. Growing evidence, in recent years increasingly fueled by deep sequencing technologies, indicates highly branched networks, complex anastomosing metabolic grids in rapidly evolving pathways. Because P450s typically catalyze irreversible reactions, they represent ideal points of control over metabolic bifurcations. In contrast to the view of P450s as highly specialized enzymes dedicated to specific pathways, evidence is emerging for substantial promiscuity of the enzymes. This may allow re-purposing and rapid evolution of novel pathways in plants and sets the stage for their combinatorial reconstitution in neo-natural pathways in biotechnology. Below we introduce the characteristics, applications and progress for each class of terpenoids individually, followed by a discussion of illustrative examples.

## Oxidative monoterpenoid metabolism

At more than 4000 known structures with over 92% carrying two, or more oxygen atoms (Dictionary of Natural Products 23.1, Pateraki et al. 2015), plant monoterpenoids show an impressive structural diversity. The short C_10_ hydrocarbon backbone, typically^1^ derived from geranyl diphosphate (GDP) establishes their physicochemical properties and, with that, their industrial applications. Limonene, for example, a simple cyclic monoterpene alkene, is used as a green solvent with a boiling point of 176 °C. A high standard enthalpy of combustion (−6100 kJ Mol^−1^) means limonene is a potential high-density biofuel. Oxygenations increase the polarity and modulate the properties and applications. Carvone or perrilyl alcohol, oxidized derivatives of limonene, have a broadened spectrum of applications, such as flavor additives or emerging cancer therapeutics (Chen et al. 2015; de Carvalho and da Fonseca 2006), emphasizing the industrial relevance of the conversion.

### Menthol

**Fig. 1:**
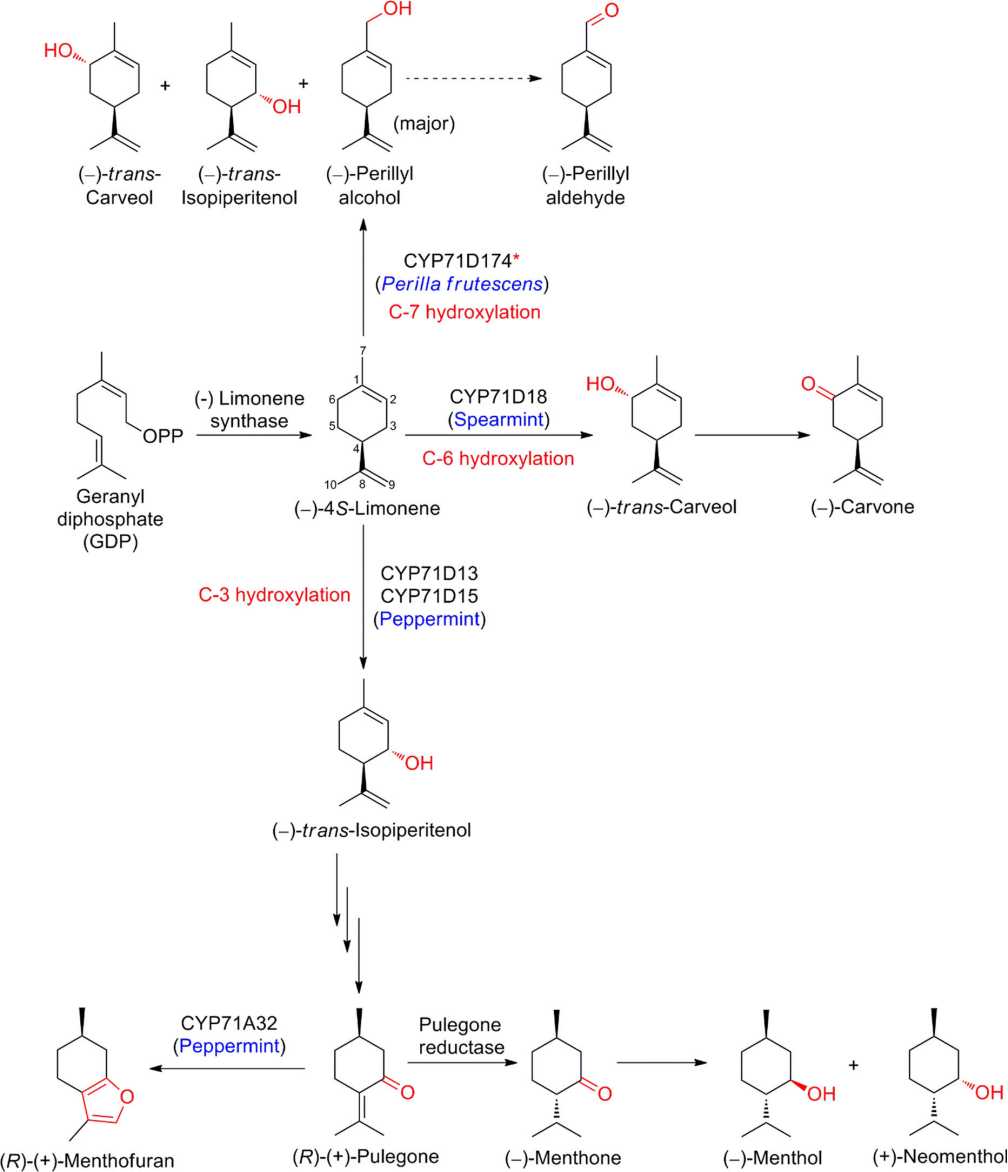
Regio-specific hydroxylation of (−)-4*S*-limonene by cytochromes P450. *Recombinant chimeric variant of CYP71D174 yields (−)-perillyl alcohol along with (−)-*trans*-isopiperitenol and (−)-*trans*-carveol in an in vitro assay

An increasing global demand of 30,000 MT/year is already exceeding current supply through novel semi-chemical strategies (e.g. BASF expects an operational (−)-menthol production unit online in 2017, https://www.basf.com/en/company/news-and-media/news-releases/2016/09/p-16-290.html, retrieved December 2016) complementing extraction from the natural sources of a growing market estimated at $300 M (Kirby and Keasling 2009). Synthetic Biology may provide an alternative to petro-based production and extraction, and biosustainable access to these natural compounds, but requires knowledge of the pathways. The essential oils from spearmint and peppermint contain oxygenated monoterpenes with characteristic and distinct positions of oxygenation on the *p*-menthane backbone. The olefinic monoterpene precursor (−)-*4S*-limonene was established as the first cyclic intermediate for the oxygenated menthanes in mint species in groundbreaking feeding studies with isotopically labelled precursors almost four decades ago in the laboratory of Rodney Croteau (Kjonaas and Croteau 1983). This opened the door for trailblazing discoveries of the molecular underpinnings of terpenoid biosynthetic pathways. Peppermint, a hybrid mint (*Mentha* × *piperita*) produces nearly exclusively C-3 oxygenated monoterpenes, e.g. (−)-menthol, whereas spearmint (*M. spicata*) accumulates C-6 oxygenated monoterpenes, e.g. (−)-carvone (overview provided in Fig. 1). In peppermint, cytochromes P450 (P450s) CYP71D15 and CYP71D13 catalyze regio-specific C-3 allylic hydroxylation of (−)-*4S*-limonene to (−)-*trans*-isopiperitenol, which is subsequently oxidized to (−)-menthol (Lupien et al. 1999; Schalk and Croteau 2000). These P450s are members of the CYP71 clan which is the major source of chemical diversification in specialized metabolism (reviewed in Hamberger and Bak 2013). Spearmint CYP71D18, on the other hand, catalyzes C-6 allylic hydroxylation of (−)-*4S*-limonene yielding (−)-*trans*-carveol, which is further oxidized to (−)-carvone (Lupien et al. 1999; Schalk and Croteau 2000). These next oxidative steps are catalyzed by members of the short-chain dehydrogenase/reductase superfamily, which are orthologous enzymes with a high degree of identity in spearmint and peppermint. The dehydrogenases cannot distinguish (−)-*trans*-isopiperitenol from (−)-*trans*-carveol as substrate (Ringer et al. 2005). Taking advantage of the regio-specificity of CYP71D15 and CYP71D18 allowed investigating the molecular basis by a combination of domain swapping and reciprocal site-directed mutagenesis. A single amino acid residue was identified controlling the regio-selectivity: exchange of the residue (F363I) in the spearmint limonene-6-hydroxylase fully converted activity and catalytic efficiency to that of the peppermint limonene-3-hydroxylase (Schalk and Croteau 2000). Thus, the closely related but functionally distinct CYP71D15 and CYP71D18 play a critical role in bifurcating the pathway from (−)-*4S*-limonene to channel the carbon flow into distinct monoterpenes in different mint species. *Perilla frutescens*, member of a different tribe within the family of Lamiaceae and, which has not undergone selective breeding for specific monoterpene profiles, accumulates the (−)-*4S*-limonene derived (−)-perillyl aldehyde in a mixture of monoterpenes in the essential oil of glandular trichomes. A partial cDNA clone homologous to the known P450s, expressed as recombinant chimeric variant CYP71D174 yielded, next to 7-hydroxy limonene (perillyl alcohol), oxidations of three of the four allylic carbons within the olefin monoterpene (Mau et al. 2010). These studies provide a classical example of monoterpene biosynthetic pathways governed by regio-selectivity of P450s. Structure–function analysis of the limonene hydroxylases inspired rational engineering of the regio-specificity of P450s by manipulation of selected residues, guided by natural variation (Lupien et al. 1999; Schalk and Croteau 2000). A complementary approach is discussed below in the paragraph for the sesquiterpenoid artemisinin, and discrete activities of members of another CYP71 subfamily.

Within the same plant system, there is a second instance of pathway control by P450s. The hepatotoxic monoterpene (*R*)-(+)-menthofuran, which also negatively affects commercial value of the essential oil, can accumulate to substantial levels under adverse growth conditions (Bertea et al. 2001). Specifically, a new P450 of the subfamily CYP71A was discovered from the peppermint oil glands. Functional characterization of heterologously expressed recombinant CYP71A32 indicated a role in formation of menthofuran by converting of (*R*)-(+)-pulegone to (*R*)-(+)-menthofuran via an allylic hydroxylation and spontaneous rearrangement yielding the furan ring (Bertea et al. 2001; Mizutani and Sato 2011). (*R*)-(+)-pulegone is also an intermediate in the route to (−)-menthol, with (+)-pulegone reductase catalyzing the conversion into the intermediate (−)-menthone (Ringer et al. 2003), effectively competing for the substrate with CYP71A32. In addition, under low-ambient light, mint plants selectively sequestered menthofuran in the oil glands at concentrations sufficient for competitive inhibition of the pulegone reductase (Mahmoud and Croteau 2001; Rios-Estepa et al. 2008). In the mint-system, improvement of the yield and purity from the natural sources was addressed by a multipronged approach, assisted by mathematical modeling: (i) overexpression of three steps of the plastidial precursor pathway provided increased isoprene C5 building blocks, (ii) a heterologous variant from a different species of the geranyl diphosphate synthase for the production of the general monoterpene precursor, geranyl diphosphate (GDP), (iii) anti-sense mediated suppression of the above menthofuran shunt-pathway, and (iv) introduction of an engineered variant of a (+)-limonene synthase. This latter feature is non-native to this system and introduces a chemical watermark permitting convenient identification of origin of the natural products of this engineered biosynthetic platform (Lange et al. 2011).

### Oxidized derivatives of linalool

**Fig. 2:**
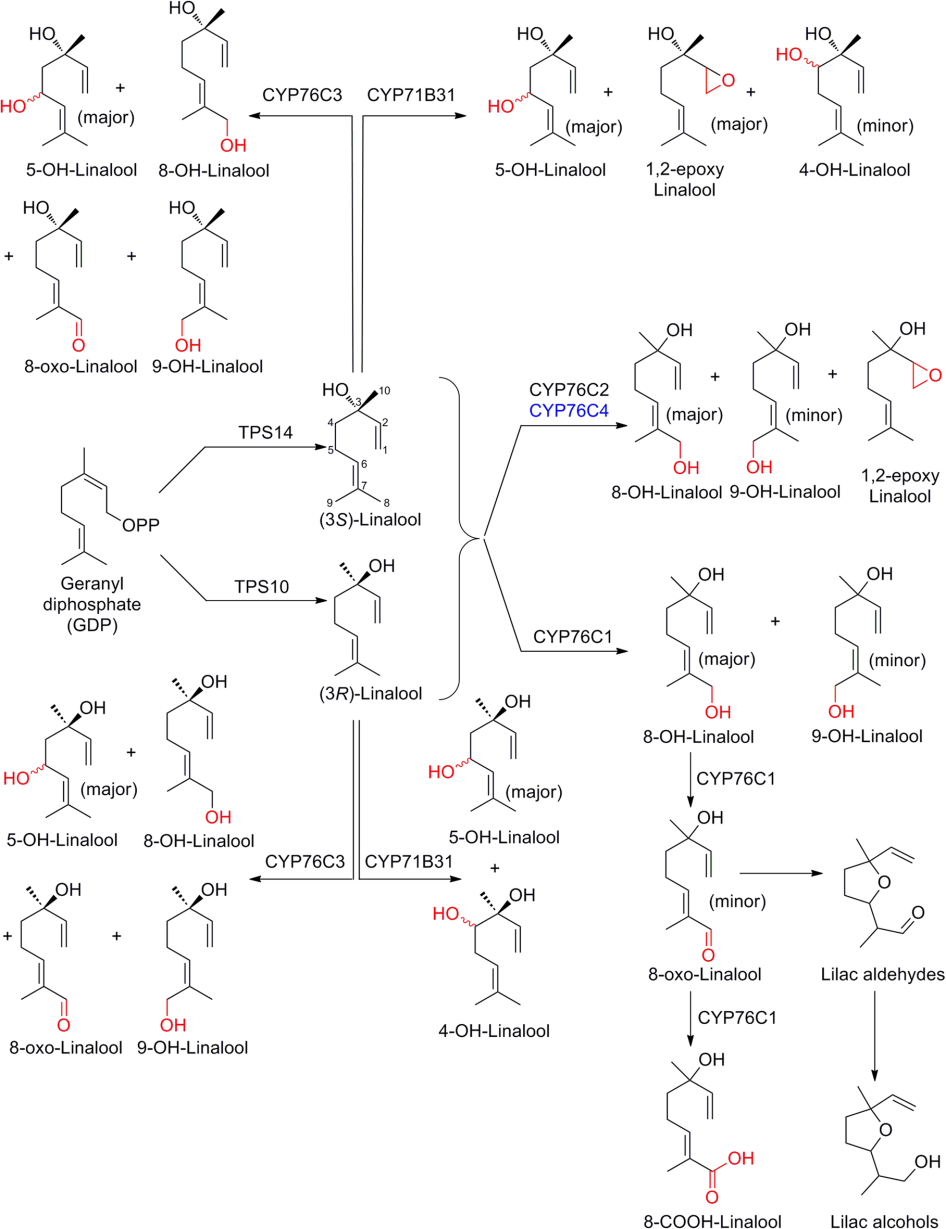
Cytochrome P450 mediated linalool metabolism in Arabidopsis. With exception of CYP76C4, the P450s are expressed in flowers. CYP76C4 has very low expression in roots. TPS: terpene synthase

Linalool is an acyclic monoterpene alcohol and contributes to the floral scents of various plants. Because of its characteristic odor, linalool is extensively used in perfumes, cosmetics, soaps, foods (Amiri et al. 2016). Linalool is synthesized from GDP by activity of the linalool synthase (TPS10 and TPS14 in *Arabidopsis*) and further oxidized by P450s, effectively generating a range of oxidized derivatives, and implying important roles for P450s in creating and directing the biodiversity of plant specialized metabolites (overview provided in Fig. 2). Two *Arabidopsis* P450s of distinct subfamilies, CYP71B31 and CYP76C3, with expression localized mainly in the flowers, were shown to catalyze oxidation of both (*R*)- and (*S*)-enantiomers of linalool to produce distinct, and partially overlapping sets of hydroxylated or epoxidized products (Ginglinger et al. 2013). From a (3*S*)-linalool substrate, CYP71B31 yielded predominantly (3*S*)-1,2-epoxylinalool and diastereomeric 5-hydroxylinalool in (3*S*,5*S*)- and (3*S*,5*R*)-configuration along with traces of (3*R*)-4-hydroxylinalool. With (3*R*)-linalool as substrate, CYP71B31 afforded the analogous diastereomeric pair of (3*R*,5*S*)- and (3*R*,5*R*)-5-hydroxylinalool as the most abundant products with (3*S*)-4-hydroxylinalool as the minor product. Interestingly, the epoxide was not formed by CYP71B31 with (3*R*)-linalool as substrate. CYP76C3 accepted both (3*S*)- and (3*R*)-linalool affording diastereomeric 5-hydroxylinalool as the major product along with 8-hydroxylinalool, 8-oxolinalool, and 9-hydroxylinalool at minor amounts (Ginglinger et al. 2013). Hence, CYP71B31 and CYP76C3 were shown to be involved in creating diversity in linalool metabolized products in *Arabidopsis*. While they displayed a lack of stereospecificity as observed for many other P450s, both were specific for linalool, as they did not accept geraniol, nerol, myrcene and ocimene among some other monoterpenes offered as substrates. In general, P450s of subfamily CYP76C act as versatile monoterpene oxidases and other members from *Arabidopsis*, CYP76C1, CYP76C2, and CYP76C4 were implicated in linalool metabolism as well, forming 8-hydroxylinalool as a major product and 9-hydroxylinalool as a minor product (Höfer et al. 2014). CYP76C4 and CYP76C2 were shown to additionally form 1,2-epoxylinalool, but unlike CYP76C3, these P450s are highly promiscuous in nature, accepting citronellol and lavandulol as substrates. CYP76C1 was found to contribute a range of multiple oxidized linalool derivatives including C-8 oxidized derivatives of linalool (8-hydroxy, 8-oxo, and 8-carboxy linalool) along with lilac aldehydes and alcohols to the floral volatile emissions of Arabidopsis (Boachon et al. 2015). Additionally, CYP76C2 and CYP76C4 were found active with nerol, CYP76C1 and CYP76C4 with α-terpineol, and CYP76C4 toward geraniol as substrates. Differential expression of the P450s across tissues and highly variable levels of transcript accumulation suggests limited functional redundancy of these genes in *Arabidopsis* (Höfer et al. 2014). This large functional spectrum of the P450s also implied a large potential for exploitation in biotechnological conversion of non-native substrates (see paragraph below on monoterpene indole alkaloids).

### Hydroxygeraniol

**Fig. 3:**
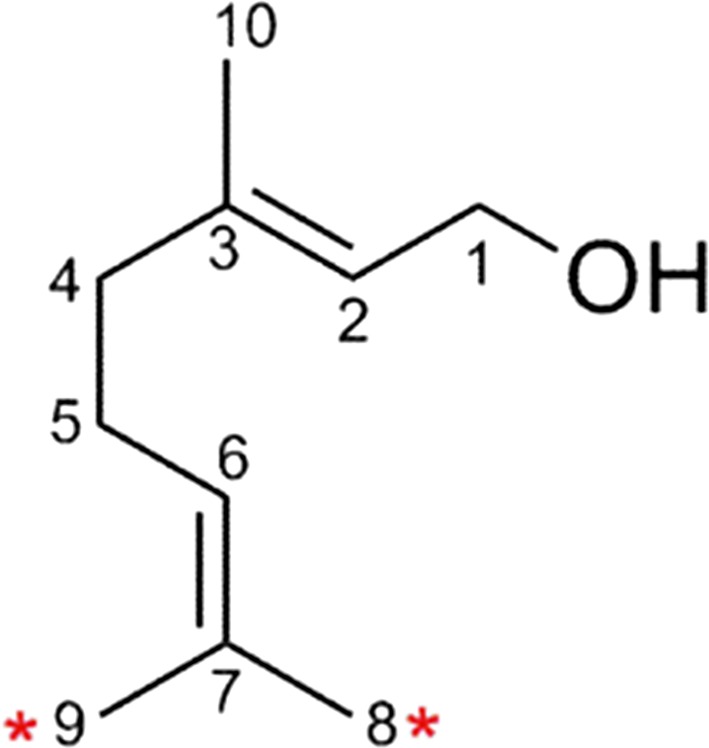
Cytochrome P450 mediated regio-specific oxidation of geraniol takes place at the marked C-8 and C-9 positions

The dialdehyde 8-oxogeranial is key intermediate in biosynthesis of the large group of monoterpene iridoids, carrying a characteristic bicyclic cyclopentane-pyrane ring system. A rare scenario for formation of the terpenoid scaffold (see paragraph below on complex macrocyclic diterpenes for a second example) is the initial oxidative activation of geraniol, a simple monoterpene alcohol by P450s and a dehydrogenase and subsequent reductive cyclization, catalyzed by the NADPH-dependent iridoid synthase (Geu-Flores et al. 2012; Kries et al. 2016). Iridoids are broadly found in plants and include monoterpene indole alkaloids, exhibiting diverse range of bioactivities (Dinda et al. 2011; Tundis et al. 2008; Viljoen et al. 2012), fueling interest for biosynthetic production. 8-hydroxylation of geraniol constitutes the first committed step in iridoid biosynthesis (Collu et al. 2001; Höfer et al. 2013). In the quest to identify optimal P450s for production of iridoid intermediates, an extended functional probing of available enzymes of the CYP76 family was undertaken. Two P450s, the archetypical CYP76B6 from *Catharanthus roseus* and CYP76C4 from *Arabidopsis* were shown to oxidize geraniol (Höfer et al. 2013). CYP76B6 catalyzed the two consecutive regio-selective C-8 oxidations to afford the aldehyde 8-oxogeraniol intermediate of the iridoid pathway, via 8-hydroxygeraniol. On the other hand, the related CYP76C4 was found to oxidize geraniol to predominantly 9-hydroxygeraniol but with 8-hydroxygeraniol only as a minor product (Fig. 3). *In vivo* reconstruction of the pathway in *Nicotiana benthamiana*, which allowed for convenient gene stacking, including CYP76C4, resulted in unspecific conversion of the generated dioxygenated 8-oxogeraniol, and to a minor extent the intermediate monoterpene diol into a range of further oxidized, reduced and conjugated derivatives (Höfer et al. 2013). This finding pointed at potential complications with this system. However, it also indicated that efficient channeling through engineered pathways can possibly outcompete endogenous, non-specific conversions, with an underlying mechanism remaining yet to be determined (see paragraph below on new perspectives).

## Diversity in sesquiterpenoid metabolism

The regio- and stereospecificity of various P450s involved in sesquiterpene biosynthesis leads to broad diversification of oxygenated sesquiterpenes. Here, our current knowledge indicates that the sesquiterpene synthases, rather than the P450s, contribute more significantly to pathway bifurcations, leading from the most common precursor of farnesyl diphosphate (FDP) to distinct families of products. However, the substrate specificity and/or promiscuity of the relevant P450s typically drive chemical diversification of the different families of sesquiterpenoids. Illustrative examples of biotechnologically motivated discovery of P450s yielding the industrially relevant sesquiterpene feedstock nootkatone and the P450 driven diversification of the structurally intriguing sesquiterpene lactones, including the flagship molecule artemisinin, have been recently reviewed (e.g. Hamberger and Bak 2013; Pateraki et al. 2015). Here we discuss selected examples of sesquiterpenes metabolite pathways, where the activity of P450s plays a key role, often at the end of the pathway.

### Santalol

**Fig. 4:**
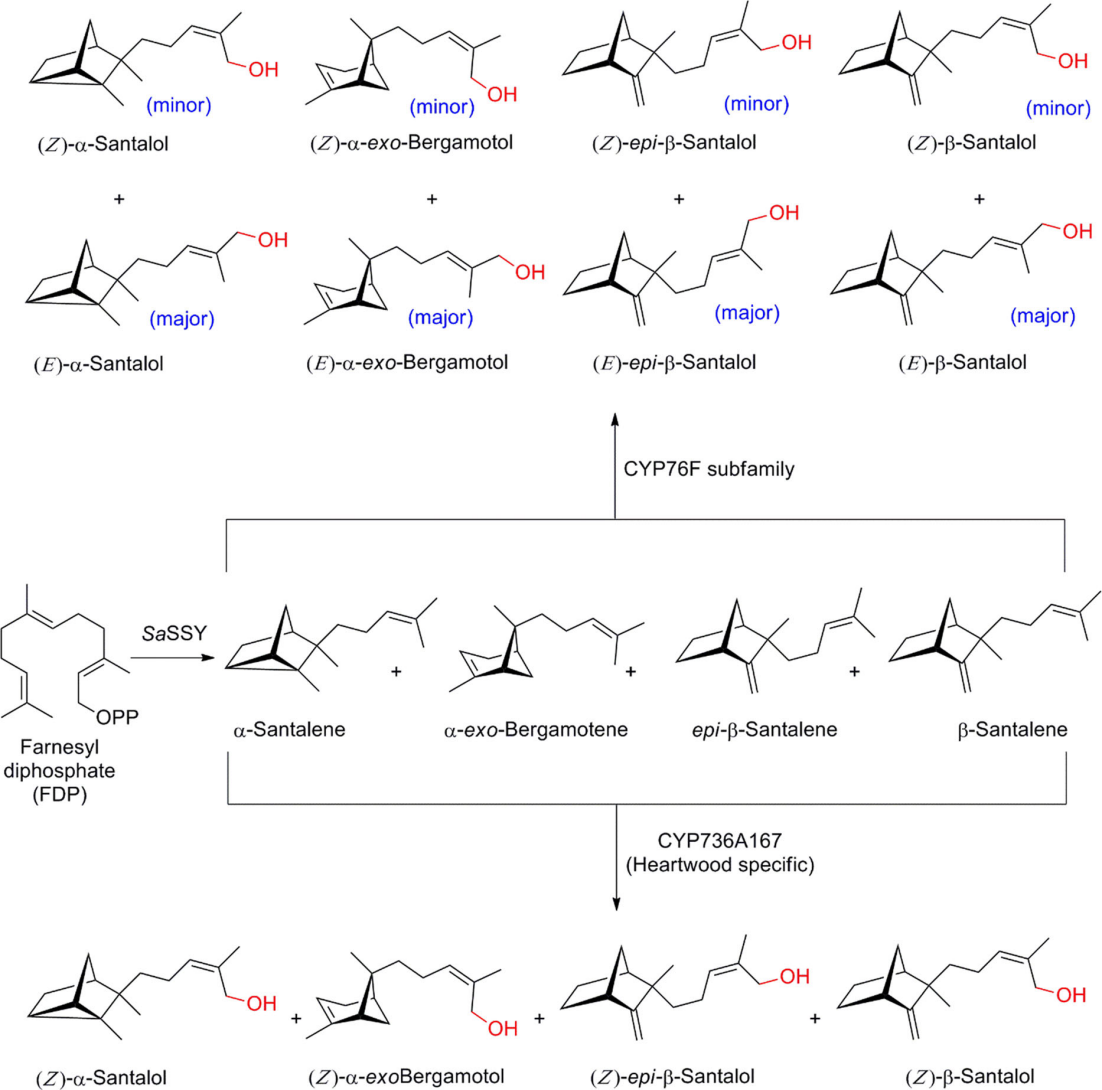
Cytochrome P450 mediated santalol metabolism in *S. album*. Stereo-specificity of heartwood specific CYP736A167 channels the olefin precursor into the (*Z*)-isomers of the constituent santalols of the sandalwood oil. *Sa*SSY: *S. album* santalene/bergamotene synthase

The sesquiterpene alcohols (*Z*)-α-santalol, (*Z*)-β-santalol, (*Z*)-*epi*-β-santalol and (*Z*)-α-*exo*-bergamotol are the predominant constituents of the essential oil of sandalwood, *Santalum ssp*. and represent high-value commercial targets for the flavor and fragrance industry, and, with a higher market volume, as bio-insecticide and insect repellent (Roh et al. 2011). Challenges in sustainable extraction from its natural source, the mature heartwood of the trees, and formal chemical synthesis have raised interest in metabolic engineering to provide alternative access to these high-value targets. A metabolic engineering strategy would require knowledge of the biosynthetic route, which has led to an ongoing line of research. A panel of orthologous sesquiterpene synthases was identified from three sandalwood species in groundbreaking work in 2011. The santalene/bergamotene synthase (SSy) catalyzes formation of a blend of α-santalene, β-santalene, *epi*-β-santalene and α-*exo*-bergamotene, which were later reported to be decorated by P450s (Celedon et al. 2016; Diaz-Chavez et al. 2013; Jones et al. 2011). Here, initial transcriptome mining of the xylem of *S. album* permitted identification of an expanded family of candidate P450 genes in the CYP71 clan. Their functional characterization established a total of ten P450s of the CYP76F subfamily (overview provided in Fig. 4; Diaz-Chavez et al. 2013). In vitro and yeast in vivo assays demonstrated that nine out of these ten genes encoded multi-substrate santalene/bergamotene oxidases with some functional redundancy. The P450s were found to hydroxylate the terminal allylic methyl group of santalenes and bergamotene to predominantly yield the (*E*)-stereoisomers (Diaz-Chavez et al. 2013). To identify the hypothesized P450 involved in formation of the sesquiterpene alcohols in (*Z*)-configuration, Celedon and co-workers integrated comparative transcriptomics across three different tissues, and critically, including the heartwood, to demonstrate a unique signature (Celedon et al. 2016). One P450 specifically fulfilled the criteria selected by the authors, of high, and spatially exclusive expression in the heartwood (Fig. 4). Intriguingly, and despite representing a member of the CYP71 clan, the candidate showed no significant homology to related enzymes of terpenoid metabolism, or the previously identified members of subfamily CYP76F, which would have rendered simple homology-informed approaches inefficient in the identification. Co-expression of codon optimized variants of *Sa*CYP736A167 and *Sa*POR2 in yeast allowed in vitro assays with supplemented substrates, while engineering of a yeast strain with the corresponding *Sa*FDPS and *Sa*SSy confirmed results by stereoselective in vivo formation of the four, main sandalwood oil sesquiterpenols in the correct (*Z*)-configuration. Here, the instructive strategy for identification of *Sa*CYP736A167 as a stereo-selective P450 in sandalwood sesquiterpene alcohols showcases its role in in vivo pathway bifurcation to channel the olefinic substrates into the specific streoisomeric alcohols, naturally present in the sandalwood oil.

### Rotundone

**Fig. 5:**
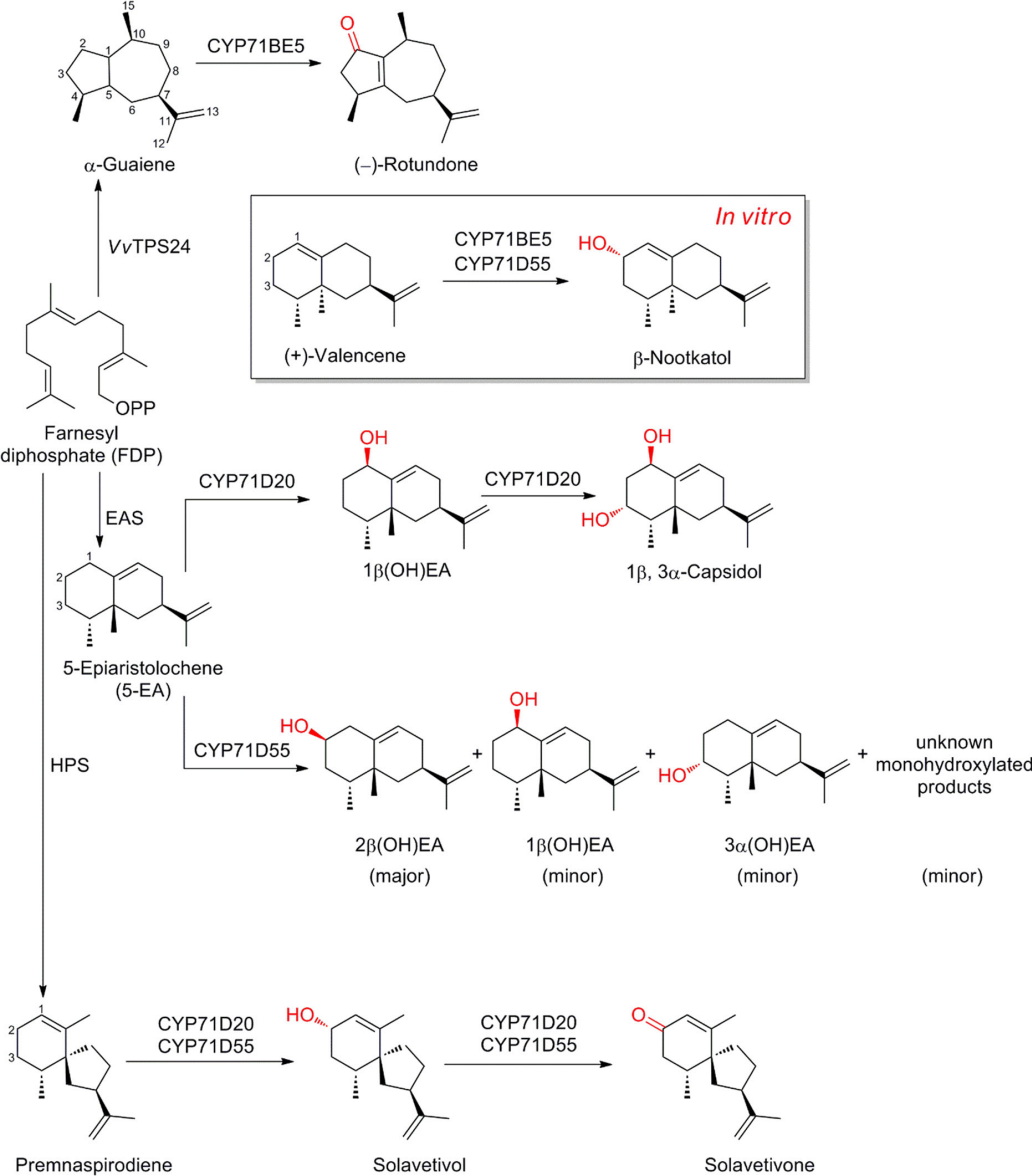
Cytochrome P450 mediated biodiversity of oxygenated sesquiterpenes in various plants. CYP71BE5 has been found to be active in Syrah grape exocarp, CYP71D20 in tobacco, CYP71D55 in henbane. *Vv*: *Vitis vinifera*, TPS: terpene synthase, EAS: 5-*epi*-aristolochene synthase, HPS: *Hyoscyamus muticus* premnaspirodiene synthase

(−)-Rotundone is an oxygenated sesquiterpene and an important aroma constituent contributing a peppery scent in various herbs and spices, including pepper, oregano and basil. Despite being present only in very low concentrations, it is also a characteristic of Shiraz wine varieties (Huang et al. 2014; Takase et al. 2016; Wood et al. 2008). The terpene synthase forming the scaffold and precursor of rotundone was identified by Drew and co-workers in the challenging system of developing grapevine (*Vitis vinifera*) berries (Drew et al. 2016). The main hurdles tackled were an expected extremely low expression of the pathway in conjunction with an unprecedented number and genomic complexity of the terpene synthase family (Martin et al. 2010). The α-guaiene synthase was found as a novel allelic variant of *VvTPS24*, established as an enzyme forming a blend of selinene-type sesquiterpenes, with only two amino acid residues controlling the product profile. Inspired by earlier reports of P450s active in terpenoid metabolism, a functional P450 of the CYP71BE subfamily was discovered as specifically expressed in the Syrah grape exocarp, consistent with accumulation of (−)-rotundone (see overview provided in Fig. 5; Takase et al. 2016). In vitro assays with recombinant enzymes in microsomes from yeast demonstrated that CYP71BE5 could also catalyze the C-2 oxidation of (+)-valencene to β-nootkatol. However, the absence of these terpenes in the Syrah grape exoxcarp suggested that CYP71BE5 functions as α-guaiene 2-oxidase *in planta*.

### Capsidol

Capsidol is a bicyclic, dihydroxylated sesquiterpene produced by many solanaceous species in response to various environmental cues including pathogen attack, elicitor challenge or exposure to UV light (Ralston et al. 2001; Takahashi et al. 2005). Biosynthesis of capsidol involves the 5-*epi*-aristolochene synthase, catalyzing formation of the bicyclic sesquiterpene olefin intermediate, 5-*epi*-aristolochene (5-EA), followed by P450-mediated dihydroxylation of 5-EA to capsidol (Facchini and Chappell 1992; Takahashi et al. 2005). The corresponding enzyme CYP71D20 from *Nicotiana tabacum* was found to catalyze the unique stereo- and regio-selective sequential dihydroxylation of 5-EA at C-1 and C-3 to afford capsidol (Greenhagen et al. 2003; Ralston et al. 2001; Takahashi et al. 2005). Investigation of the kinetic behavior of CYP71D20 established the putative sequence of oxidation of 5-EA at the C-1 position followed by the C-3 position, generating stereoselectively 1*β*-hydroxylated EA followed by 1*β*,3*α*-capsidol (Takahashi et al. 2005). CYP71D20 has also been found to catalyze the conversion of premnaspirodiene to solavetivone, albeit at very low rates (Greenhagen et al. 2003). Although the pathway bifurcation for the sesquiterpene hydrocarbon intermediates 5-EA and premnaspirodiene depends on the corresponding sesquiterpene synthase, the stereo-and regio-selective catalysis by CYP71D20 of the individual sesquiterpene hydrocarbon intermediate plays an important role in creating the diversity of the oxygenated sesquiterpenes (Fig. 5).

### Solavetivone

Solavetivone, a potent antifungal phytoalexin, plays a role as defense molecule in the solanaceous plants henbane (*Hyoscyamus muticus*) and potato. It is biochemically synthesized from the vetispirane-type sesquiterpene based scaffold premnaspirodiene, which is formed by the *H. muticus* premnaspirodiene synthase. Another member of the CYP71 subfamily, CYP71D55, was found to catalyze the successive regio-selective oxidation at the carbon atom C-2 position of premnaspirodiene to yield solavetivone (Fig. 5). In vitro assays demonstrated that CYP71D55 also converted valencene and 5-EA with an eremophilane based sesquiterpene scaffold, but only to their corresponding mono-oxygenated product (Takahashi et al. 2007), unlike the related P450s catalyzing successive oxidations such as CYP71D20 and CYP71AV8 (Cankar et al. 2011; Ralston et al. 2001). As with previous examples, CYP71D55 is involved in increasing the biodiversity of the oxygenated sesquiterpenes.

### Artemisinin

The founding member of the subfamily, CYP71AV1, from *Artemisia annua* catalyzes a three-step oxidation of amorphadiene to artemisinic acid, off-product of the pathway to the anti-malaria pharmaceutical artemisinin (Ro et al. 2006). The homology-based identification of orthologous sequences in closely related Asteraceae led to the discovery of enzymes with lowered regioselectivity (i.e. formation of a distinct amorphadienol isomer), in addition to a lack of activity for the formation of artemisinic acid (Komori et al. 2013). The initial number of different amino acids over the entire sequence was narrowed down to a section carrying nine residues through functional testing of chimeric variants with swapped domains. Using structural modeling, four amino acids emerged, putatively residing in the catalytic site. Following site directed mutagenesis and functional testing, conversion of a serine to phenylalanine in position 479 reduced the conversion of the alcohol to the aldehyde nearly 20-fold and limited the activity of the P450 to a single step (Komori et al. 2013). While not of immediate biotechnological relevance, as the pathway to Artemisinin proceeds via the aldehyde, this approach highlights the rational engineering of the activity of a P450 with an important role at this metabolic junction, guided by natural variation and structural modeling.

## Oxidation of labdane-type and macrocyclic diterpenes

With backbones consisting of 20 carbon atoms, the theoretically possible structural complexity of diterpenes far exceeds that of the classes using two or three C5 building blocks. Yet, among documented plant terpenoids, the number of diterpenoids is in the same range of sesquiterpenoids, with substantially fewer monoterpenoids known (diterpenoids, 12,505 vs. sesquiterpenoids 13,981 vs. monoterpenoids, 4129; Dictionary of Natural Products 23.1; Pateraki et al. 2015). On the other hand, over 95% of known diterpenoid structures are carrying two or more oxygen atoms (sesquiterpenoids, 90%; monoterpenoids, 92%), indicating increased relevance of P450s in generation of structural complexity and chirality in the respective biosynthetic routes.

**Fig. 6:**
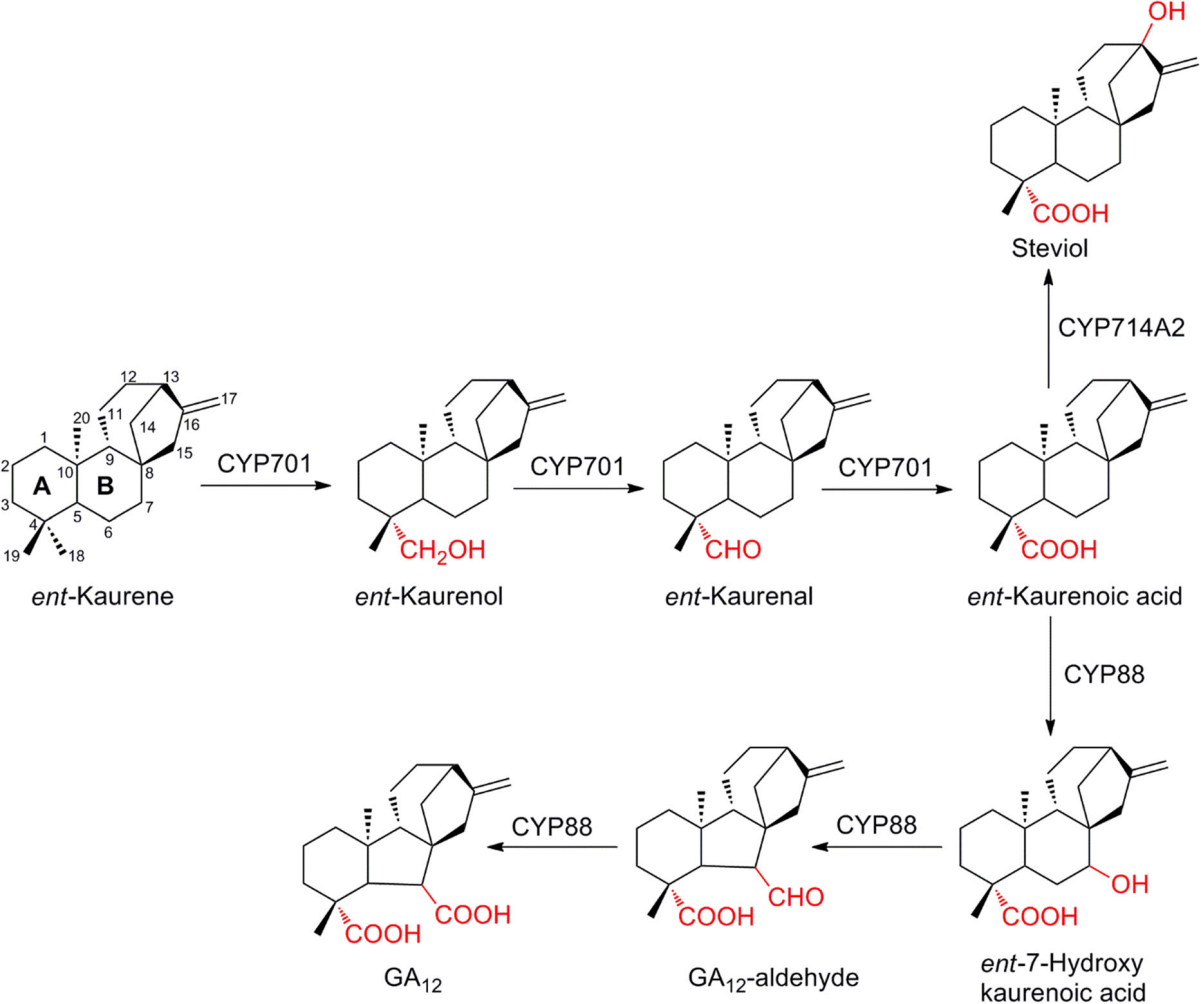
Cytochrome P450 mediated regio-specific oxidation of *ent*-kaurene in GA_12_ biosynthesis. Regio-specificity of P450s (CYP714A2 and CYP88) leads to pathway bifurcation between general and engineered specialized metabolism

Gibberellins (GAs) are archetypal plant diterpenes and hormones involved in the regulation of plant growth and development, but also controlling developmental processes such as germination, flowering, and reproduction. Formation of GA_12_, the simplest of the GAs, is catalyzed in a linear route by two classes of enzymes, a pair of diterpene synthases (diTPS) cyclize *ent*-kaurene from the common acyclic precursor geranylgeranyl diphosphate (GGDP) and a pair of sequentially acting P450s oxidizing *ent*-kaurene via *ent*-kaurenoic acid to GA_12_ (Helliwell et al. 2001). The P450s involved are noteworthy, as they are members from two divergent families, CYP701 and CYP88, which, after duplication, have repeatedly served as starting material for the evolution of enzymes in specialized metabolism. The concept of P450s as drivers of the evolution of specialized metabolism has been reviewed in Hamberger and Bak, (2013), while a plant- and biotechnological perspective for their discovery is given in (Nelson and Werck-Reichhart 2011; Pateraki et al. 2015), respectively. CYP701 is the only member of the CYP71 clan known to be involved in terpenoid general metabolism and catalyzes the regio-specific three-step oxidation at carbon atom C-18 of *ent*-kaurene to *ent*-kaurenoic acid (Helliwell et al. 1999). CYP88 is found in the CYP85 clan and catalyzes further oxidation and contraction of the B-ring of *ent*-kaurenoic acid to GA_12_ (see overview provided in Fig. 6; Helliwell et al. 2001). Combinatorial biosynthesis, which assembles both natural and neo-natural pairs of diTPS into modules has been used to biosynthesize a highly diverse array of diterpene scaffolds (Andersen-Ranberg et al. 2016). This approach was used to probe the activity of two representative members of mono- and dicotyledon CYP701A with 22 different labdane-type diterpenes (Mafu et al. 2016). *At*CYP701A3 was found to exhibit a very broad promiscuity and accepted two-thirds of the diterpenes, while the rice ortholog *Os*CYP701A6 was strictly specific towards *ent*-kaurene and *ent*-isokaurene. It was speculated that this phenomenon correlates with the accumulation of diverse labdane-type diterpenoids in rice, but not in Arabidopsis, and consequently requires higher specificity in rice (Mafu et al. 2016). Next to possible implications for enzyme evolution, this study was among the first probing the promiscuity of plant P450s with terpenoids with an outstanding relevance in biotechnological applications (see below another example, biosynthesis of steviol). Dedicated P450s of terpenoid specialized metabolism in rice have been discovered in multiple families, expanded in the CYP71 clan, and present an intriguing diversity. Their biochemical activity and involvement in control of the pathways to bioactive diterpenoids of the labdane-class has been comprehensively reviewed (Schmelz et al. 2014). Recently, *ent*-10-oxodepressin, a novel casbane-type macrocyclic diterpene phytoalexin was discovered in rice (Inoue et al. 2013). This indicates that, despite our substantial understanding gained since characterization of the first P450 in phytoalexins biosynthesis more than two decades ago (Kato et al. 1995), additional pathways remain to be discovered.

### Casbene based diterpenes

**Fig. 7:**
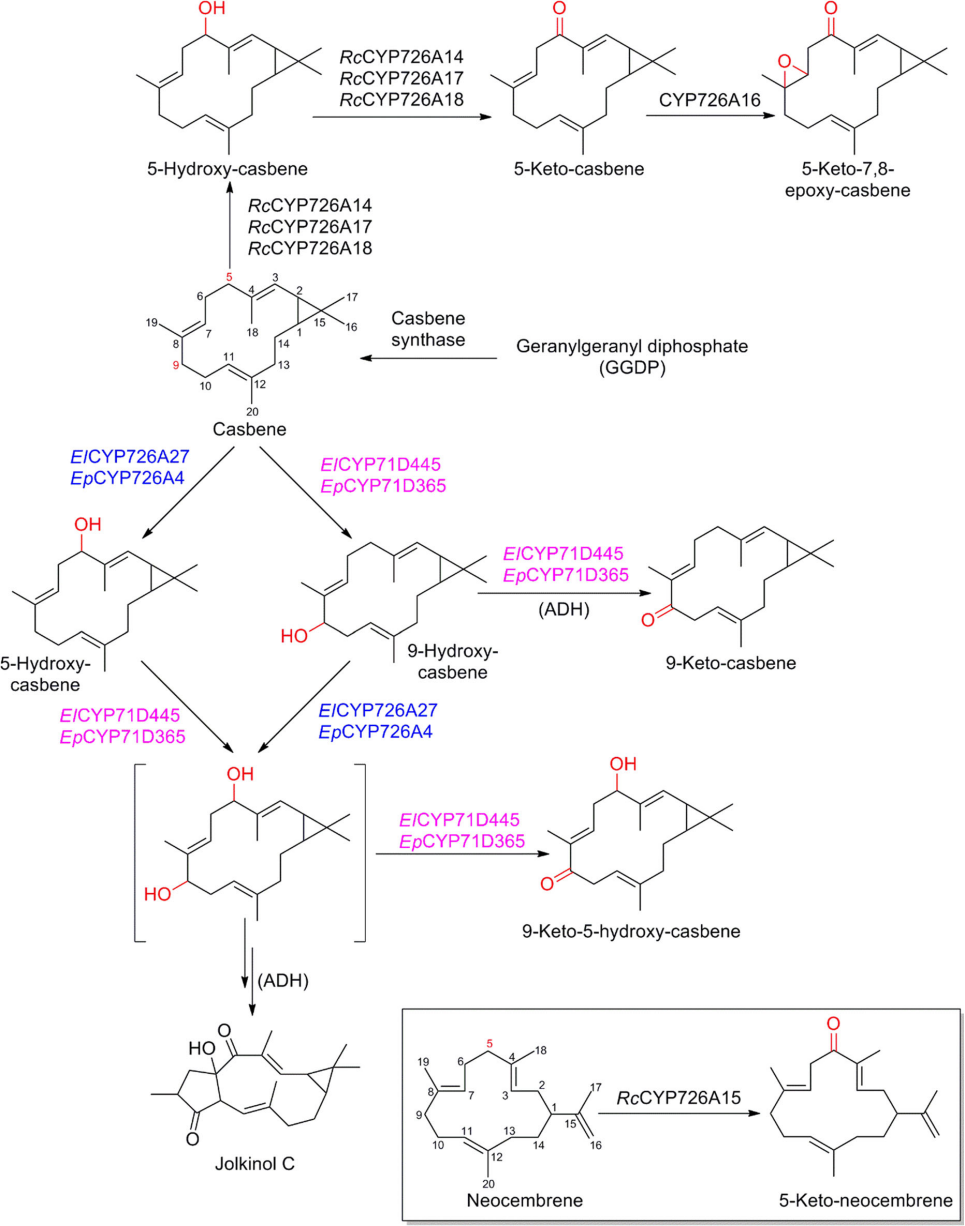
Regio-specific oxidation of casbene by different orthologous members of cytochromes P450. *ADH* alcohol dehydrogenase

Casbene (and related cembrene/neocembrene) and casbene derived phorbol ester diterpenoids increase in complexity with additional ring closures to become lathyrane, jatrophane, tigliane and ingenol, and their derivatives. Together they constitute characteristic macrocyclic diterpenoids of the Euphorbiaceae family (Appendino 2016). Their pharmaceutical applications include anti-cancer, anti-viral and anti-tumor activities. The structural diversity of these compounds results from an unconventional biosynthetic route, requiring activation of the backbone of the simple precursor macrocyclic diterpene by P450s prior to additional cyclization and re-arrangement (Luo et al. 2016). The principle resembles formation of iridoid terpenoids, where the reductive cyclization was shown to entail activation of the monoterpene scaffold (Geu-Flores et al. 2012) in contrast to typical diterpenoid biosynthetic routes, where the cyclization represents the first committed entry step of the pathway. Mining for candidate P450s, a significant, Euphorbiaceae-specific bloom in subfamily CYP726A of tribe 71D (consisting of subfamily CYP71D and additional closely related subfamilies) was reported earlier in *E. peplus*, which accumulates ingenane diterpenoids. Inspired by an activity of the founding member from *E. lagascae* in formation of structurally unusual epoxy-fatty esters (CYP726A1, Cahoon et al. 2002), a function of CYP726A members in phorbol ester formation was proposed (Zerbe et al. 2013). In the Euphorbiaceae castor bean (*Ricinus communis*) it was shown that C-5 oxidation of casbene is catalyzed by CYP726A14, CYP726A17 and CYP726A18 (King et al. 2014, Boutanaev et al. 2015). 5-keto-casbene, characteristic of simpler bicyclic diterpenes in that species, was found to be further epoxidized by CYP726A16 (see overview provided in Fig. 7). The genes encoding these P450 were reported clustered in the genome of castor bean with the corresponding casbene synthase. A shared pattern of co-expression, which implied a role in the pathway to oxidized casbenes *in planta* facilitated their discovery (King et al. 2014). Another member of the same subfamily CYP726A15, which is also located in the same gene cluster in castor bean, was found to be active on neocembrene forming 5-keto-neocembrene. Recently, two casbene-activating P450s of sub-families CYP71D445 and CYP726A27 were isolated from *E. lathyris* (Luo et al. 2016), which allowed identification of functional orthologues also from *E. peplus* (CYP71D365 and CYP726A4 respectively). Key for their discovery was analysis of the mature seed transcriptome of *E. lathyris*, which, in contrast to the earlier developed transcriptome in *E. peplus*, was highly enriched in full length sequences of P450s in those families. Functional characterization, both in the transient *N. benthamiana* system and engineered yeast demonstrated that CYP71D445 and CYP71D365 catalyzed the regio-specific C-9 oxidation of casbene. Considering that this specific oxidized position is characteristic of complex multicyclic diterpene types, it was proposed that this activity controlled an early bifurcation steps in the biosynthesis of macrocyclic diterpenoids. Combining CYP71D445 with CYP726A27 and CYP71D365 with CYP726A4 yielded oxidation at C-5 and formation of 9-keto casbene and 9-keto-5-hydroxy-casbene, mechanistically implausible intermediates to multicyclic diterpenes. Indeed, in conjunction with an enzyme of the alcohol dehydrogenase family, co-expressed with the casbene synthase and P450s, it was demonstrated that instead of the oxidized ketone derivatives, hydroxyl derivatives of casbene were effectively converted to the multicyclic jolkinol C (Luo et al. 2016). Notably, engineered strains of yeast for production of casbene and expression of the P450s were critical for independent confirmation of the enzyme activities and for isolating intermediates supporting the hypothesized pathway. However, these strains did not afford jolkinol C, indicating that potential limitations of the current yeast system will need to be addressed for further engineering of biosynthetic production towards higher cyclized phorbol esters.

### Carnosic acid, tanshinones, steviol and forskolin

**Fig. 8:**
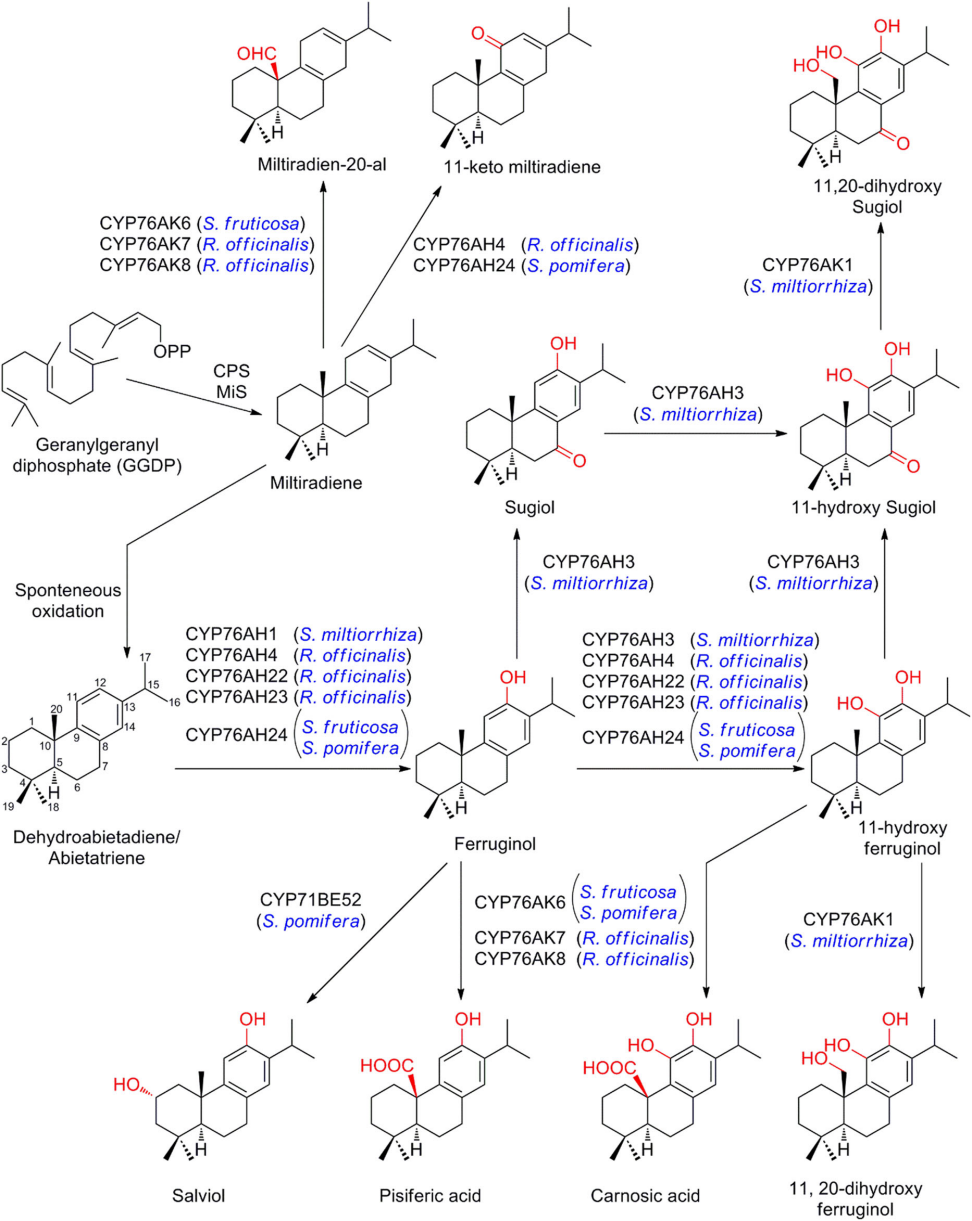
Regio-specific oxidation of miltiradiene by different orthologous members of cytochromes P450. Regio-specificity of P450s leads to pathway bifurcation and metabolic diversity. *CPS* copalyl diphosphate synthase, *MiS* miltiradiene synthase

Labdane-type diterpenoids are the most widely distributed type of diterpenoids in specialized metabolism (Zi et al. 2014). Carnosic acid and its related derivatives are diterpenes with a distinctive aromatic ring and exhibit a wide range of activities including antioxidant, anticancer and antimicrobial activities (Ignea et al. 2016; Scheler et al. 2016). These compounds belong to the group of labdane-type diterpenes with a bicyclic decalin core and are formed through the intermediates of miltiradiene, dehydroabietadiene (or abietatriene) and ferruginol. The P450s involved in the biosynthesis of carnosic acid have recently been identified from *Salvia pomifera, Rosmarinus officinalis,* and *S. fruticosa* (overview provided in Fig. 8; Ignea et al. 2016; Scheler et al. 2016). Scheler and co-workers reported in elegant work that *Ro*CYP76AH4, *Ro*CYP76AH22, *Ro*CYP76AH23, *Sf*CYP76AH24 from *R. officinalis* and *S. fruticosa* carry out two successive oxygenations of dehydroabietadiene to form ferruginol and 11-hydroxyferruginol, expanding on earlier reports of homologs which only yielded ferruginol (Bozic et al. 2015, see also below). Modeling studies and site directed mutagenesis established that individual amino acid residues in the active site confer specificity of the P450s towards either formation of ferruginol, or in case of the multifunctional enzymes, both formation of ferruginol and its further oxidation to 11-hydroxy ferruginol (CYP76AH4, CYP76AH22 to CYP76AH24). This work also identified three members of a closely related subfamily, *Sf*CYP76AK6, *Ro*CYP76AK7, and *Ro*CYP76AK8 from *S. fruticosa* and *R. officinalis* as oxidases with a specificity for carbon atom C-20, and that accept both ferruginol and 11-hydroxyferruginol as well as miltiradiene to produce pisiferic acid, carnosic acid and miltiradien-20-al, respectively (Scheler et al. 2016). Elucidation of the complete biosynthetic pathway for carnosic acid/pisiferic acid and the rational site-directed mutagenesis of the required P450s to modulate their specificity is encouraging for future metabolic engineering of these phenolic diterpenes in microbial hosts.

Independent, complementary research found that CYP76AH24, CYP76AK6 from *S.*- *pomifera* and CYP76AH4, CYP76AK8 from *R. officinalis* account for the set of oxidations in the pathway from dehydroabietadiene to carnosic acid (Ignea et al. 2016). Functional analysis of the P450s in engineered yeast showed in addition that yet another member of a related subfamily, CYP71BE52, can oxidize ferruginol in the C-2 position to salviol. Hence, CYP76AH22/CYP76AH24/CYP76AH4, CYP76AK6/CYP76AK7/CYP76AK8, and CYP71BE52 control multiple pathway bifurcations leading to chemical diversification in the biosynthesis of dehydroabietadiene based diterpenoid metabolism.

*Salvia miltiorrhiza*, another member of the family of Lamiaceae, is well known for accumulating tanshinones, which have expansive uses in traditional Chinese medicine, but having also attracted interest due to anti-bacterial and a range of therapeutic activities. Tanshinones, like carnosic acid, are abietane-type diterpenoids, derived from ferruginol and through a biosynthetic route involving members of subfamilies CYP76AH and CYP76AK: founding members CYP76AH1 and CYP76AK1 of both subfamilies were discovered in *S*. *miltiorrhiza*. CYP76AH1 was established as ferruginol synthase, however, a rather obscure mechanism was proposed to explain the apparent conversion of the diterpene olefin miltiradiene to ferruginol (Guo et al. 2013). This misperception was later corrected when the orthologous CYP76AH4 from *R. officinalis* was functionally characterized demonstrating that ferruginol is produced by CYP76AH4 mediated oxidation of dehydroabietadiene (abietatriene), which is a spontaneous oxidation product from miltiradiene (Zi and Peters 2013). Subsequent identification and characterization of CYP76AH3 and CYP76AK1 by expression in yeast led to demonstration of a pathway with at least one intersection and bifurcation controlled by these P450s. CYP76AH3 catalyzed the oxidation to yield a C-11 hydroxyl function, as well as formation of 7-keto ferruginol (sugiol) and 7-keto-11-hydroxyferruginol (11-hydroxy sugiol). In contrast, CYP76AK1 showed regio-selectivity for oxidation at carbon atom C-20 of both 11-hydroxy ferruginol and 11-hydroxy sugiol (Guo et al. 2016).

Another group of labdane-type diterpenoids with commercial relevance are steviol-glucoside sweeteners accumulating in the leaves of the Asteraceae *Stevia rebaudiana*. These are based on a bifurcation, leading from the GA_12_ intermediate *ent*-kaurenoic acid through a single hydroxylation at carbon atom C-13 to steviol (Fig. 6). Recombinant Arabidopsis CYP714A2, with a role in GA metabolism, was shown to also yield steviol, when incubated with *ent*-kaurenoic acid (Nomura et al. 2013). Inspired by this finding, an active *ent*-kaurenoic acid hydroxylase was identified in *S. rebaudiana*. An engineered variant of CYP714A2 finally yielded over 15 mg L^−1^ of steviol, when expressed in a strain of *E. coli* dedicated for production of *ent*-kaurenoic acid (Fig. 6; Wang et al. 2016).

In the Lamiaceae *Coleus forskohlii*, diterpenes carrying both abietane and epoxy-labdane (13*R*-manoyl oxide) scaffolds are prevalent, and their biosynthetic origins have been elucidated (Pateraki et al. 2014). Of interest in this plant species is the root-specific accumulation of the 13*R*-manoyl oxide-derived structurally complex diterpene forskolin, which consists of an oxygen heteroatom-containing labdane scaffold, with five functionalized positions. Identification of a substantial bloom of subfamily CYP76AH yielded an intriguing number of enzymes with, in part multifunctional activity towards 13*R*-manoyl oxide and, concomitant, extensive chemical diversification of the product palette. Ultimately, combinatorial testing led to a minimal set sufficient to catalyze regio- and stereo-specific formation of deacetyl-forskolin. Isolation of a regio-selective acetyl-transferase completed a biosynthetic route to forskolin, which was stably integrated in an engineered yeast strain optimized for production of 13*R*-manoyl oxide (Pateraki et al. 2017; patents Andersen-Ranberg and Pateraki 2016; Hamberger et al. 2015, 2016).

### Diterpene Resin Acids

**Fig. 9:**
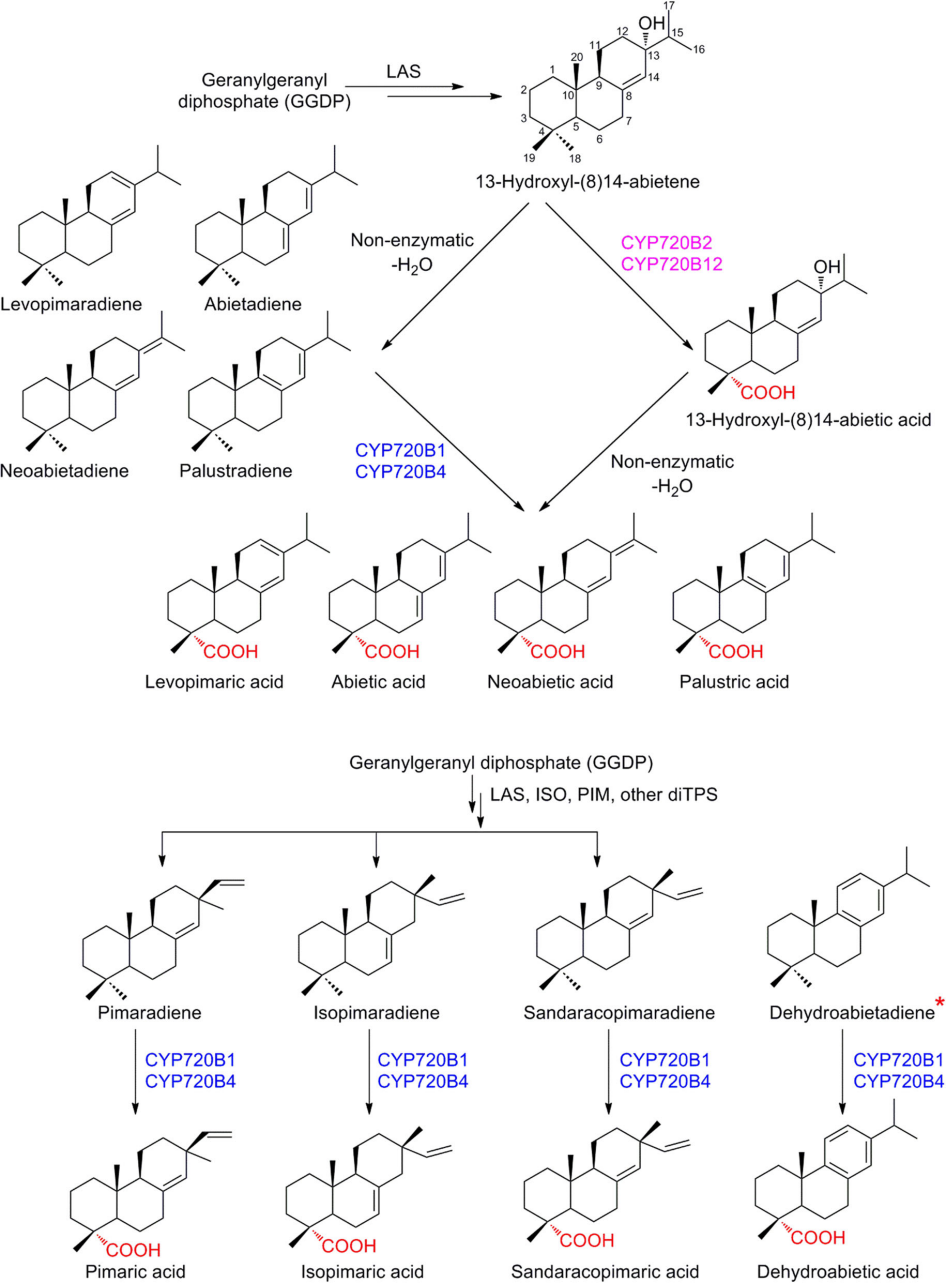
Cytochrome P450 mediated regio-specific oxidation of various diterpene scaffolds leads to chemical diversity of diterpene resin acids in conifers. Example of P450s from different clades in subfamily CYP720B which yield the same products through distinct routes. *The pathway forming dehydroabietadiene from GGDP is not known in conifers. *LAS* levopimaradiene/abietadiene synthase, *ISO* isopimaradiene synthase, *PIM* pimaradiene synthase, *diTPS* diterpene synthase

The labdane-type diterpene resin acids are important constituents of the oleoresin defense of conifers and have both constitutive and induced protective roles for protection of these long-lived plants against fast evolving pests and pathogens. Industrial applications include the use of resin acids as biopolymers, constituents of high-end inks, in glues, tackifiers and as coatings (Bohlmann and Keeling 2008). The sequential activities of diterpene synthases (diTPSs) and cytochromes P450, creating a mixture of chemically divergent resin acids, and to a lesser extent aldehydes and alcohols, is increasingly well understood and may serve as illustrative example of meticulous research providing deep insights into the genetic and biochemical underpinning of chemical diversity. The bifunctional diTPS catalyzing stereo-specific formation of (+)-abietadiene and the corresponding P450-related activity have been known for over two decades, with the first P450 cloned and characterized in 2005 (Funk and Croteau 1994; LaFever et al. 1994; Ro et al. 2005). Formation of the backbones of four distinct diterpenes through the activity of the Norway spruce (*Picea abies*) diTPS appeared established, until Keeling and co-workers demonstrated that the initial enzyme product is the thermally labile tertiary alcohol 13-hydroxy-8(14)-abietene, which, after water elimination yields the known mix of four olefins, levopimaradiene, abietadiene, neoabietadiene and palustradiene (Keeling et al. 2011). Similarly, for the P450s, established members of CYP720B subfamily, *Pinus taeda* (loblolly pine) CYP720B1 and *Picea sitchensis* (Sitka spruce) CYP720B4 from the CYP85 clan, were shown to oxidize a range of diterpene olefins to yield the corresponding resin acids *in planta*, with production in engineered yeast reaching 0.9 and 0.2 mg L^−1^ for isopimaric acid and abietic acid, respectively (see overview provided in Fig. 9, Hamberger et al. 2011; Ro et al. 2005). These enzymes are members of a largely expanded subfamily with about a dozen P450s in each conifer species investigated. Recently, a comprehensive investigation by Geisler and co-workers provided evidence for this impressive genetic diversity and the missing link with the unstable diterpene product. CYP720B2 and CYP720B12, members of a distant clade of the previously established P450s were functionally characterized in three conifer species, *Pinus banksiana* (jack pine), *P. contorta* (lodgepole pine) and *P. sitchensis*. While the enzymes did not accept diterpene olefins, they efficiently catalyzed regio-selective C-18 oxidation of abietaenol, converting the unstable diterpene alcohol (13-hydroxyl-(8)14-abietene in Fig. 9) into the corresponding hydroxyl-resin acid, ultimately yielding after non-enzymatic water loss, the panel of levopimaric acid, abietic acid, neoabietic acid and palustric acid. In contrast, the diterpene olefin isopimaradiene, which does not proceed via a tertiary alcohol during biosynthesis is effectively oxidized by CYP720B4 and CYP720B1, but not the enzymes accepting the diterpene alcohol as substrate, highlighting the exceptional modularity and plasticity of conifer diterpene metabolism (Geisler et al. 2016). The distinct difference in the substrate specificity of the P450s results in an alternate and extended but finally convergent route for conifer resin acid biosynthesis, with reconnected end products of both distant clades. From a biotechnological perspective, those multifunctional P450s have a high potential in synthetic pathways when combined with diterpene modules. For example, CYP720B4 has recently been used to engineer the in vivo production of dehydroabietic acid from glucose via miltiradiene in yeast, which was hypothesized as a key intermediate en route to the diterpene therapeutic triptolide from *Tripterygium wilfordii* (Forman et al. 2017).

### Paclitaxel

**Fig. 10:**
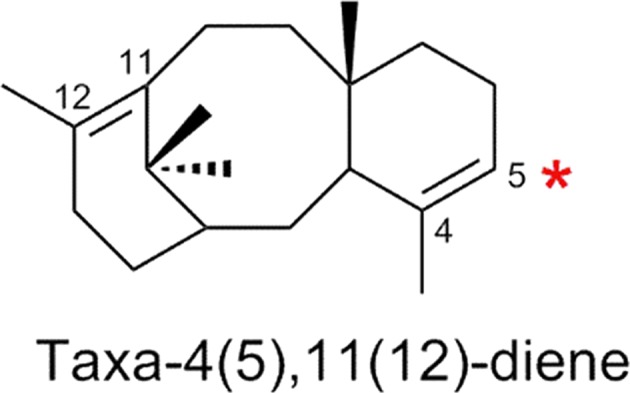
CYP725A4 mediated regio-specific oxidation of taxadiene takes place at the marked C-5 position

Paclitacel (Taxol^®^), a highly functionalized cancer therapeutic, and arguably one of the highest-value diterpenoids, is found in the bark of yew (*Taxus* ssp.) species (Wani et al. 1971). Due to the low accumulation in its natural host (i.e. 0.02% yield from the concentrated alcohol extract of the stem bark of *Taxus brevifolia*; Wani et al. 1971), the biotechnological production of paclitaxel has captured the interest of scientists for a long time to complement production in cell culture (Howat et al. 2014). The biosynthesis of paclitaxel was estimated to involve approximately 20 discrete enzymatic steps (Chau et al. 2004). The first committed step is formation of taxa-4(5),11(2)-diene, the macrocyclic core skeleton of paclitaxel (Wildung and Croteau 1996). Based on the prevalent distribution of the pattern of hydroxylation, the next predicted step requires the P450 taxadiene-5α-hydroxylase, CYP725A4, which catalyzes the regio-selective hydroxylation of the 5α position of taxadiene to yield taxa-4(20),11(12)-dien-5α-ol (T5OH/T-5α-ol) (Fig. 10, Biggs et al. 2016b; Edgar et al. 2016; Hefner et al. 1996; Jennewein et al. 2004). The subsequent conversions include a series of P450-mediated oxygenations, providing the molecular handles for acylations and further conjugation of the skeleton. These include in a yet not fully elucidated sequence of carbon atoms at C-2, C-9, C-10, C-13 and C-14 (Jennewein and Croteau 2001; Jennewein et al. 2003, 2001; Schoendorf et al. 2001; Walker and Croteau 2001). Much to the detriment of approaches building biosynthetic platforms, even the early steps show metabolic bifurcations. For example, acetylation of T5OH was found to re-route the taxoid into further hydroxylation at carbon C-10, or C-14 by two P450s, while the free alcohol T5OH underwent oxidation at carbons C-9, or C-13, depending on the P450 (reviewed in Kaspera and Croteau 2006). Potential multifunctional enzymes and uncertainties of the sequence of conversions dramatically complicated the rational design of routes to individual products. Hence, substantial effort was spent investigating the established first oxidative step, which is highly illustrative for the challenges encountered. Various research groups have described the heterologous expression and biosynthetic formation of taxa-4(5),11(12)-diene and its first oxidation by CYP725A4 in chassis organisms like yeast, tobacco, *E. coli*, tomato (Ajikumar et al. 2010; Dejong et al. 2006; Engels et al. 2008; Huang et al. 2001; Kovacs et al. 2007; Rontein et al. 2008). Heterologous production of taxadiene was achieved in substantial amounts, and oxidation, resulting in further products was accomplished to a minor degree (Zhou et al. 2015). However, metabolic engineering efforts showed that the formation of the CYP725A4-mediated 5-hydoxylated product is highly limited due to a behavior of this enzyme possibly dependent on the experimental context. It has been observed that the heterologous expression of taxadiene synthase and CYP725A4 in tobacco (*Nicotiana sylvestris*) result in the formation of 5(12)-oxa-3(11)-cyclotaxane (OCT) instead of T5OH (Rontein et al. 2008). Further studies described that expression of recombinant taxadiene synthase and CYP725A4 in an *E. coli* host leads to equal formation of T5OH and OCT and indicated that the lack of specificity of the oxygenation represents a first limitation to overcome (Ajikumar et al. 2010; Edgar et al. 2016; Zhou et al. 2015). Investigating the mechanism more in detail, earlier studies have suggested that promiscuous H-atom abstraction from both the isomeric olefinic precursors, taxa-4(5),11(2)-diene and taxa-4(20),11(2)-diene, forming an allylic radical followed by oxygen insertion can lead to the formation of T5OH (Hefner et al. 1996; Jennewein et al. 2004). On the other hand, two independent recent studies have suggested an epoxide intermediate (Barton et al. 2016; Edgar et al. 2016) and formation of a mixture of products derived from taxa-4(5),11(2)-diene and a single oxidation product from taxa-4(20),11(2)-diene, concluding that an unstable epoxide intermediate may contribute to the native product profile of CYP725A4 (Edgar et al. 2016). In contrast, and supporting promiscuity of CYP725A4, Biggs and co-workers performed in vivo characterization in engineered *E. coli* and in vitro characterization in a nanodisc platform, confirming the formation of T5OH, OCT, iso-OCT and additional oxygenated taxoids at titers exceeding 500 mg L^−1^ (Biggs et al. 2016a). This example of paclitaxel may well reflect two other high profile scenarios, underscoring the challenges met by Synthetic Biology in production of industrially relevant targets at commercially competitive level: artemisinin and morphine (Oye et al. 2015; Peplow 2016).

## Oxidative pathways of triterpene specialized metabolism

**Fig. 11:**
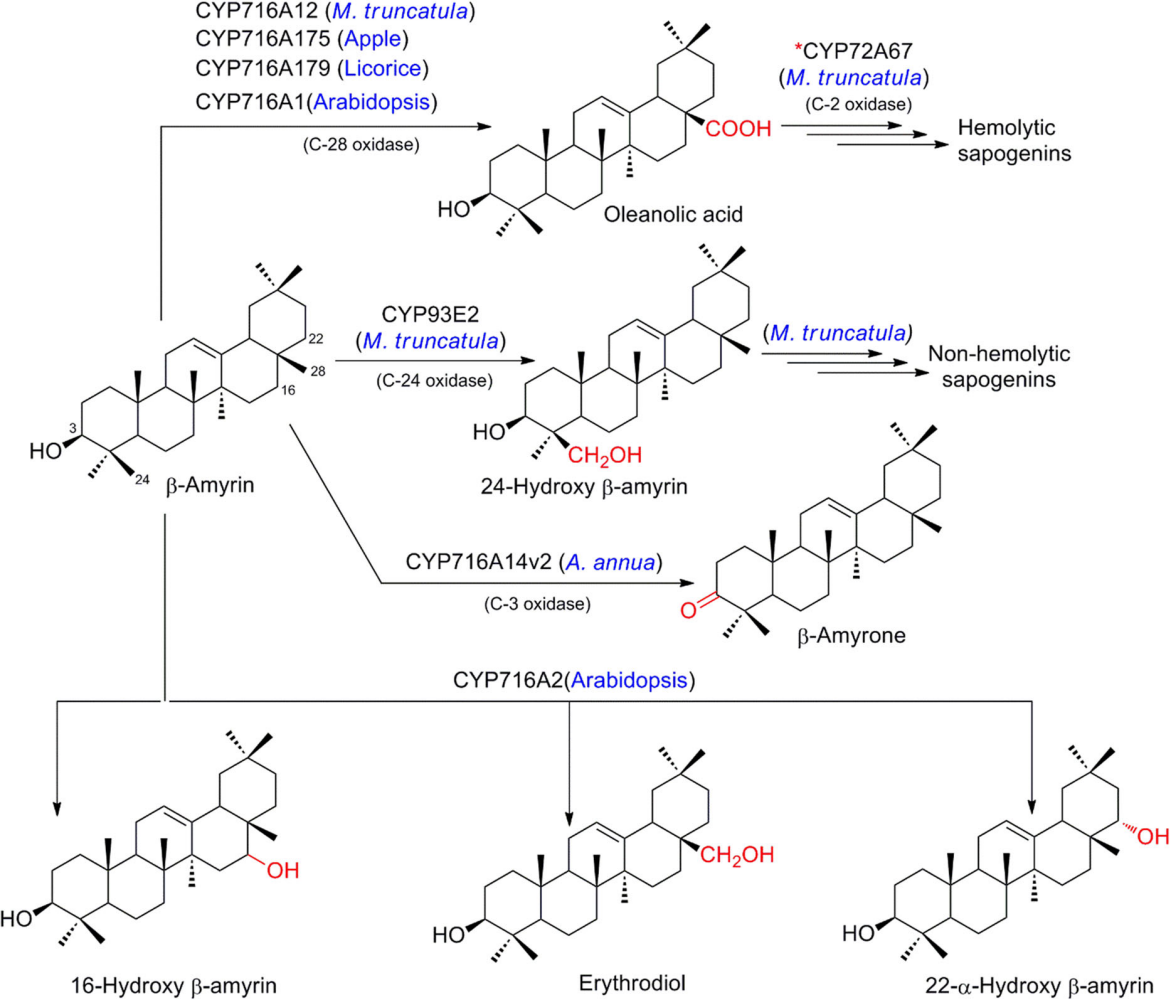
Regio-specific oxidation of β-amyrin by different orthologous members of cytochromes P450. *Key cytochrome P450s controlling the regio-specific C-2 oxidation of various intermediates involved in sapogenin biosynthesis

**Fig. 12:**
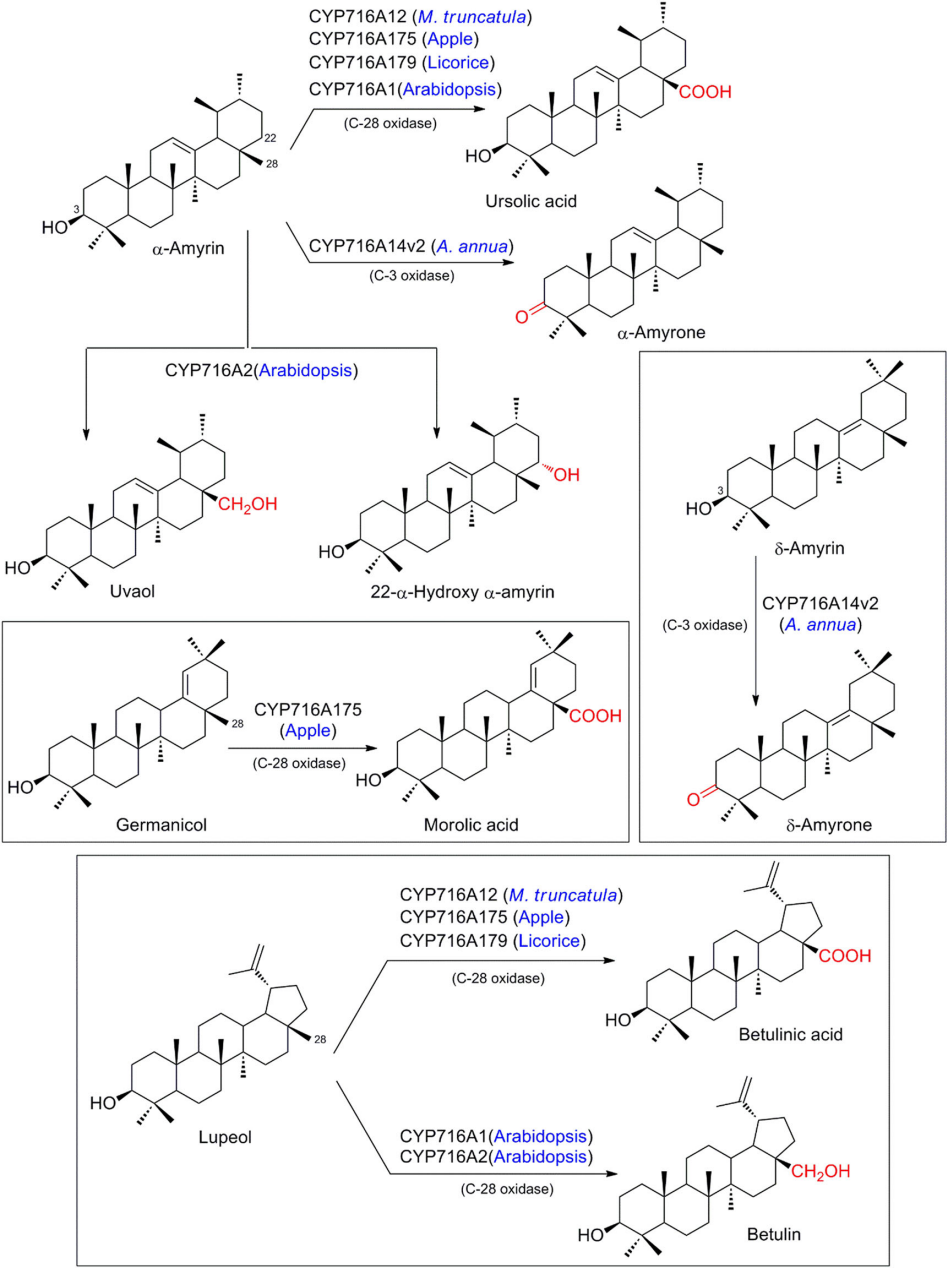
Regio-specific oxidation of α-amyrin, δ-amyrin, germanicol, and lupeol by various orthologous members of cytochromes P450

Saponins are a diverse group of sugar conjugated triterpenes detected in a broad variety of plants including medicinal plants such as licorice and ginseng, as well as crop plants, such as legumes and oats (Fukushima et al. 2011; Haralampidis et al. 2002). Biotechnological interest in saponins is fueled by applications in the pharmaceutical and agrochemical industries, but also their use in food and cosmetics (Huhman et al. 2005; Sparg et al. 2004; Suzuki et al. 2002; Tava and Avato 2006). Our knowledge of oxidative functionalization of the triterpene scaffold by P450s and the subsequent glycosylation has dramatically expanded during the last decade, and since the first enzyme with activity towards both β-amyrin and sophoradiol was identified in soybean (Shibuya et al. 2006). An excellent overview of the history of discovery and the diverse reactions of triterpenoids catalyzed by P450s and corresponding glucosyl transferases was recently published by Seki and co-workers (Seki et al. 2015). Based on this substantial body of evidence, it is well established that regio- and stereospecific oxidations by P450s in triterpenoid metabolism represent critical points of divergence controlling subsequent modification and conjugation. For their illustrative nature, we briefly provide an example of regiospecific gatekeepers controlling the biosynthetic pathways of two types of saponins from β-amyrin, and further focusing on the most recent developments and studies of biotechnological relevance (see overview provided in Figs. 11 and 12).

### Recruitment of highly diverse P450s for triterpenoid oxidation

Studies involving selection of candidate genes by co-expression analysis in *Medicago truncatula* followed by their in vivo and in vitro functional characterization demonstrated that CYP716A12 acts as β-amyrin 28-oxidase (Carelli et al. 2011; Fukushima et al. 2011; Naoumkina et al. 2010). CYP716A12 catalyzes three sequential oxidation steps at C-28 position of β-amyrin to produce oleanolic acid, which gets further decorated by other P450s to produce hemolytic sapogenins. In transgenic yeast, CYP716A12 is also shown to oxidize α-amyrin and lupeol to ursolic acid and betulinic acid, respectively (CYP716A175 and CYP716A179 from apple and licorice, respectively, have identical activity, Fig. 11). On the contrary, CYP93E2 from *M. truncatula* has been found to oxidize β-amyrin at C-24 forming 24-hydroxy β-amyrin (and also probably β-amyrin-24-oic acid) which further leads to the biosynthesis of non-hemolytic sapogenins (soyasapogenols) (Fukushima et al. 2011). This highlights the recruitment of highly divergent P450s from different clans for controlling the metabolic junction in these routes and has implications for pathway discovery driven by identification of recent expansions of gene families. Specifically, CYP93E2 is a member of the notoriously enriched CYP71 clan, where numerous functions in terpenoid specialized metabolism have spawned. In contrast, CYP716A12 resides in the CYP85 clan, with broader involvement in terpenoid general metabolism, but which also carries evolutionarily old examples of specialized metabolism such as in the conifer lineage (Kaspera and Croteau 2006; Ro et al. 2005). In recent advances, *M. truncatula* CYP72A67 was identified through TILLING in a mutagenized population. Through functional characterization by genetic approaches and heterologous expression, CYP72A67 was established in the context of other related P450s as the key enzyme controlling oxidation at C-2 of several intermediates in the hemolytic sapogenin pathway to zhantic acid (Biazzi et al. 2015). This body of work, together with numerous studies in other plant systems, solidly established the P450 families CYP93, CYP716 and CYP72 as rich repositories for candidates involved in tripterpenoid oxidation. However, isolated examples have also occurred in other families. This has inspired recent studies, where since 2015 members of CYP716 were implicated as key enzymes in C-28 oxidation of α-amyrin, β-amyrin, and lupeol leading to the corresponding acids in apple (see overview provided in Fig. 12, CYP716A175; Andre et al. 2016), and analogously in licorice, producing ursolic acid, oleanolic acid, and betulinic acid (CYP716A179, Tamura et al. 2017). Similar oxidation is also observed for germanicol to morolic acid by CYP716A175. In *Artemisia annua*, best known for the elucidated biosynthesis of the sesquiterpenoid artemisinin, CYP716A14v2 was shown to catalyze C-3 oxidation of α-amyrin, β-amyrin, and δ-amyrin to yield the 3-keto triterpenes (Moses et al. 2015). Founding members of the subfamily, Arabidopsis CYP716A1 and CYP716A2, were reported in a genomic cluster co-localized on chromosome 5 with a triterpene synthase. When co-expressed with the triterpene synthase, CYP716A1 displayed activity and afforded an oxidized tirucalla-7,24-dien-3β-ol (Boutanaev et al. 2015). Comprehensive testing of both P450s in yeast, engineered for production of α-amyrin, β-amyrin, and lupeol established them as multifunctional enzymes, with partially overlapping functions. CYP716A1 catalyzed multiple oxidations at specific positions of the tripterpene scaffolds toward ursolic acid and oleanolic acid but not betulinic acid. In contrast, CYP716A2 was limited to a mono-oxidation, yielding the alcohol intermediates of the triterpenes, 22-α-hydroxy α-amyrin and traces of β-amyrin oxidized at C-28 and C-16. With that, CYP716A2 contributes triterpene oxidation of carbon C-22 to the existing toolbox enabling combinatorial biosynthesis (Yasumoto et al. 2016). Formation of additional, yet unidentified oxidized triterpenes by other relatives in subfamily CYP716A (Khakimov et al. 2015) and broad phylogenetic distribution of P450s with demonstrated activity in triterpenoid oxidation over at least eight subfamilies in four clans (Yasumoto et al. 2016) highlights a promising biosynthetic potential for biotechnological production of high-value triterpenoids.

### Triterpenoid sweeteners

**Fig. 13:**
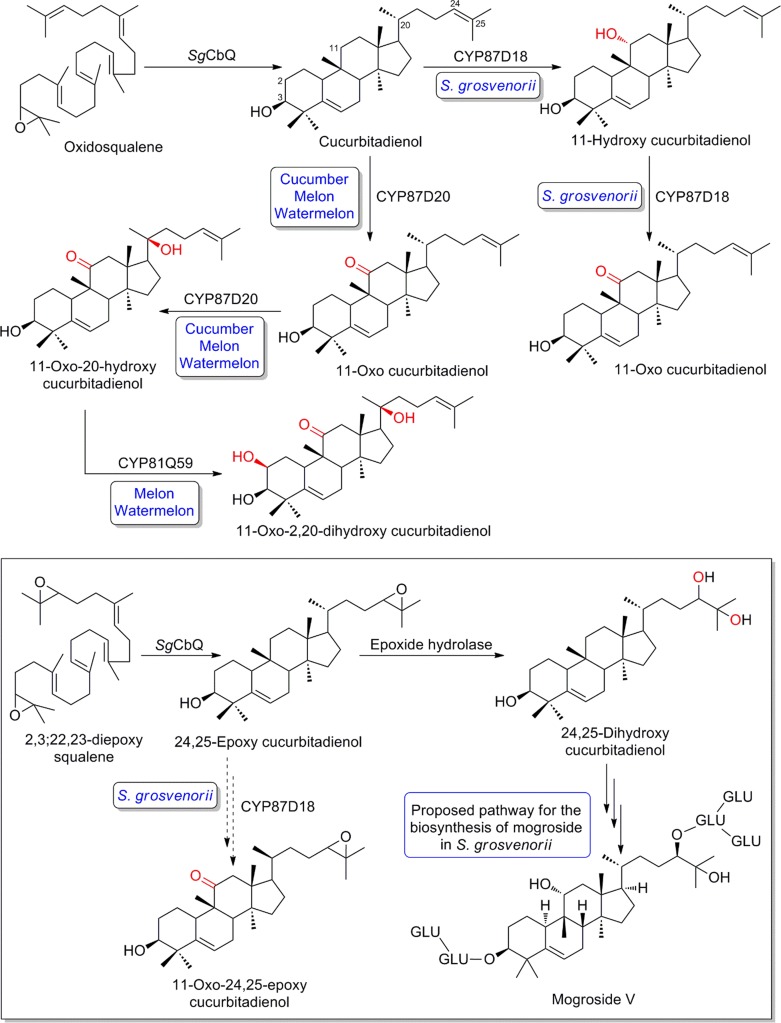
Regio-specificity of orthologous members of cytochromes P450 in the metabolism of cucurbitadienol. *SgCbQ S. grosvenorii* cucurbitadienol synthase

From a biotechnological perspective, triterpene saponins have attracted considerable interest for their therapeutic activity, and as non-sugar sweeteners. The pathway to glycyrrhizin, the main sweet-tasting triterpenoid saponin found in the roots of Chinese licorice (*Glycyrrhiza* ssp.), is well understood. Involvement of CYP88D6 as an 11-oxidase of β-amyrin in the glycyrrhizin biosynthetic pathway has been established and was comprehensively reviewed recently (Seki et al. 2008, 2015). Another group of triterpenoid based sweeteners which has been focus of intense research and seen substantial recent progress are mogrosides, accumulating in the Chinese cucurbit *Siraitia grosvenorii* (Cucurbitaceae). Mogrosides carry the scaffold of the cucurbitane triterpenoids, broadly distributed in the family, including the bitter cucurbitacins with a wide palette of pharmaceutical activities. In the biosynthetic route to cucurbitacins and mogrosides, cyclization of the general triterpenoid precursor oxidosqualene to cucurbitadienol was suggested to represent the first committed step, but alternative routes may exist (see below). Distinguishing feature of the different classes of bioactive cucurbitanes is a characteristic pattern of oxidative decoration of the tetracyclic scaffold. In a burst of recent advances, and elegantly integrating knowledge of genomic clustering of the key elements of the pathway, co-expression analysis and comparative genomics of closely related cucurbits, considerable light was shed by several teams on the evolution of the routes, including the governance P450s exert (see overview provided in Fig. 13, Itkin et al. 2016; Shang et al. 2014; Zhang et al. 2016; Zhou et al. 2016). Specifically, orthologous members of subfamily CYP87D, residing in the CYP85 clan, were discovered in *Siraitia*, cucumber, melon and watermelon. The P450s were shown to yield 11-oxo cucurbitadienol when combined with the triterpene synthase cucurbitadienol synthase in engineered yeast strains. In addition, 11-oxo-24,25-epoxy cucurbitadienol was found to accumulate, along with the monohydroxylated 11-hydroxy cucurbitadienol specifically in *Siraitia* (Zhang et al. 2016). The further oxidized 11-oxo-20-hydroxy cucurbitadienol was detected in cucumber, melon and watermelon, while co-expression with CYP81Q orthologs from melon and watermelon specifically yielded 11-oxo-2,20-dihydroxy cucurbitadienol (Zhou et al. 2016). This particular activity could not be confirmed in cucumber. Instead, cucumber and *Siraitia* carry members of the divergent subfamily CYP88L, yielding 19-hydroxy derivatives of cucurbitadienol, consistent with saponins found in these species (Itkin et al. 2016; Shang et al. 2014).

Reconstruction of a complete mogroside recombinant pathway, including optimization of production and several steps catalyzed by UDP-dependent glycosyl transferases, has been reported (Liu et al. 2014). However, the origin of the vicinal C-24 and C-25 hydroxyl groups in the mogroside aglycon remained unclear with alternatives possible (Itkin et al. 2016). C-24 and C-25 hydroxyl groups are rare among cucurbitane triterpenoids. Zhang and co-workers proposed an origin of the intermediate epoxide through activity of CYP87D18 (Zhang et al. 2016). In contrast, based on observations in yeast and transient expression in tobacco, where endogenously formed 2,3;22,23-diepoxy squalene is offered as substrate for the cucurbitadienol synthase, Itkin and co-workers suggest an initial di-epoxydation of squalene, and cyclization to 24,25-epoxy cucurbitadienol as plausible intermediate step. Hydrolytic ring opening to yield the vicinal 24,25-diol was suggested to be catalyzed by epoxide hydrolases and not to require activity of P450s (for an overview see Fig. 13; Itkin et al. 2016).

## New perspectives

### Regulation and organization

Two recent studies shed light on a previously unrecognized level of metabolic regulation and organization. Parage and co-workers suggest that control of the monoterpene indole alkaloid pathway in *C. roseus* and activity of the large group of P450s involved, including CYP76B6, is administrated through the enzyme providing the reduction equivalents, the NADPH-dependent cytochrome P450 oxidoreductase (POR, synonym CPR) (Parage et al. 2016). With exceptions (Apiaceae, Andersen et al. 2016), PORs are typically represented by two distinct classes. Specifically, *in planta*, but not in reconstituted or in vitro systems, a member of the *C. roseus* class II POR was shown to be essential for the specialized metabolism responding to external stimuli. In contrast, the class I POR was found to be associated with the general metabolism, with no measurable contribution in the metabolism of monoterpene indole alkaloids, as shown by deep co-expression and silencing studies (Parage et al. 2016). Further highlighting a critical mechanistic importance of the POR in specialized metabolism, a recent study of the protein–protein interaction in context of the local lipid environment demonstrated the existence of a dynamically assembled and disassembled metabolon (Laursen et al. 2016). The team led by Jean-Étienne Bassard proposed an operative stabilization of the metabolon when all enzymes are co-expressed and interact. The work establishes a higher complexity order, including homo- and heterodimers of the POR, a soluble glycosyl transferase, and the two involved P450s, resulting in a highly efficient biosynthetic pathway. Despite using an experimental model outside of terpenoid metabolism, it is suggested that the principle of organization in dynamic metabolons may apply more broadly to biosynthetic pathways involved in specialized metabolism (Laursen et al. 2016). This principle may also have implications for the rational engineering of orchestrated pathways or scaffolded complexes in Synthetic Biology, where a critical goal is to enable effective channeling of intermediates while avoiding disadvantageous metabolic shunt pathways, leakage of labile intermediates or unspecific endogenous activities in the chassis organism.

### The chassis

The insights discussed in this review highlight only a few of the complexities encountered during the engineering of biotechnological production platforms, and when depending on heterologous enzymes which plausibly evolved to drive chemical diversification in their plant source species. On the other hand, the biotechnological host species, or chassis, presents conceptual challenges, which need to be overcome (reviewed in Renault et al. 2014). The use of heterotrophic microbes has been comprehensively reviewed (e.g. Li and Pfeifer 2014) and efficient gene-stacking of P450s remains a limitation. A recent study elegantly demonstrated the production of di-oxygenated and acetylated taxadiene through a synthetic consortium of both *E. coli* and *S. cerevisiae*. The novel approach successfully engineered interdependency between the species and split the pathway into two segments, each expressed in one of the microbes (Zhou et al. 2015). Photosynthetic hosts offer several potential advantages, including presence of reducing equivalents (electrons) derived from photosynthesis and a source of carbon. Even though there is currently no photosynthetic platform with reported scaled and stable production of multiple oxidized terpenoids at industrially relevant level, recent proof-of-concept studies in cyanobacteria, algae, the lower land plant *Physcomitrella patens* and chloroplast engineering (reviewed in Nielsen et al. 2016) highlight the potential for these platforms. Finally, vascular land plants have evolved highly specialized anatomical structures dedicated for terpenoid storage, such as glandular trichomes, laticifers and resin ducts. This structural repertoire was recently suggested to include lipid droplets for storage of both simple and highly functionalized terpenoids (Pateraki et al. 2014). As terpenes were shown to potentially cause critical perturbations on the thermotropic and structural properties of lipid bilayers (Jagalski et al. 2016), the coordinated engineering of both terpenoid biosynthetic pathways and the formation of intracellular storage organelles may relieve this potential bottleneck.

## Footnotes

1 For monoterpenes, GDP (the *trans*-isomer/*E*-isomer) is the most common precursor, although the *cis*-/*E*-isomer, neryl diphosphate was also reported in *Solanaceae*, but with terpene synthases accepting the substrate also outside the family. Similarly, the prenyl diphosphate in *trans*-configuration *E,E*-farnesyl diphosphate (FDP) and *E,E,E*-geranylgeranyl diphosphate (GGDP) are the most common precursors for sesqui- and diterpenes, respectively. The corresponding *cis*-isomers, i.e. all-*cis Z,Z*-farnesyl diphosphate and nerylneryl diphosphate (*Solanaceae*), can also serve as precursors for some sesqui- and diterpene synthases (Jones et al. 2011; Matsuba et al. 2013; Schilmiller et al. 2009).

## References

[CR1] Ajikumar PK, Xiao W-H, Tyo KEJ, Wang Y, Simeon F, Leonard E, Mucha O, Phon TH, Pfeifer B, Stephanopoulos G (2010). Isoprenoid pathway optimization for Taxol precursor overproduction in *Escherichia coli*. Science.

[CR2] Amiri P, Shahpiri A, Asadollahi MA, Momenbeik F, Partow S (2016). Metabolic engineering of *Saccharomyces cerevisiae* for linalool production. Biotechnol Lett.

[CR3] Andersen TB, Hansen NB, Laursen T, Weitzel C, Simonsen HT (2016). Evolution of NADPH-cytochrome P450 oxidoreductases (POR) in Apiales—POR 1 is missing. Mol Phylogenet Evol.

[CR4] Andersen-Ranberg J, Pateraki E (2016) Biosynthesis of oxidised 13R-MO and related compounds. Google Patents; WO2016070885A1

[CR5] Andersen-Ranberg J, Kongstad KT, Nielsen MT, Jensen NB, Pateraki I, Bach SS, Hamberger B, Zerbe P, Staerk D, Bohlmann J, Moller BL, Hamberger B (2016). Expanding the landscape of diterpene structural diversity through stereochemically controlled combinatorial biosynthesis. Angew Chem Int Ed.

[CR6] Andre CM, Legay S, Deleruelle A, Nieuwenhuizen N, Punter M, Brendolise C, Cooney JM, Lateur M, Hausman JF, Larondelle Y, Laing WA (2016). Multifunctional oxidosqualene cyclases and cytochrome P450 involved in the biosynthesis of apple fruit triterpenic acids. New Phytol.

[CR7] Appendino G (2016). Ingenane diterpenoids. Prog Chem Org Nat Prod.

[CR8] Barton NA, Marsh BJ, Lewis W, Narraidoo N, Seymour GB, Fray R, Hayes CJ (2016). Accessing low-oxidation state taxanes: is taxadiene-4(5)-epoxide on the taxol biosynthetic pathway?. Chem Sci.

[CR9] Bertea CM, Schalk M, Karp F, Maffei M, Croteau R (2001). Demonstration that menthofuran synthase of mint (*Mentha*) is a cytochrome P450 monooxygenase: cloning, functional expression, and characterization of the responsible gene. Arch Biochem Biophys.

[CR10] Biazzi E, Carelli M, Tava A, Abbruscato P, Losini I, Avato P, Scotti C, Calderini O (2015). CYP72A67 catalyzes a key oxidative step in *Medicago truncatula* hemolytic saponin biosynthesis. Mol Plant.

[CR11] Biggs BW, Lim CG, Sagliani K, Shankar S, Stephanopoulos G, Mey MD, Ajikumar PK (2016). Overcoming heterologous protein interdependency to optimize P450-mediated Taxol precursor synthesis in *Escherichia coli*. Proc Natl Acad Sci USA.

[CR12] Biggs BW, Rouck JE, Kambalyal A, Arnold W, Lim CG, De Mey M, O’Neil-Johnson M, Starks CM, Das A, Ajikumar PK (2016). orthogonal assays clarify the oxidative biochemistry of taxol P450 CYP725A4. ACS Chem Biol.

[CR13] Boachon B, Junker RR, Miesch L, Bassard JE, Hofer R, Caillieaudeaux R, Seidel DE, Lesot A, Heinrich C, Ginglinger JF, Allouche L, Vincent B, Wahyuni DS, Paetz C, Beran F, Miesch M, Schneider B, Leiss K, Werck-Reichhart D (2015). CYP76C1 (Cytochrome P450)-mediated linalool metabolism and the formation of volatile and soluble linalool oxides in arabidopsis flowers: a strategy for defense against floral antagonists. Plant Cell.

[CR14] Bohlmann J, Keeling CI (2008). Terpenoid biomaterials. Plant J.

[CR15] Boutanaev AM, Moses T, Zi J, Nelson DR, Mugford ST, Peters RJ, Osbourn A (2015). Investigation of terpene diversification across multiple sequenced plant genomes. Proc Natl Acad Sci USA.

[CR16] Bozic D, Papaefthimiou D, Bruckner K, de Vos RC, Tsoleridis CA, Katsarou D, Papanikolaou A, Pateraki I, Chatzopoulou FM, Dimitriadou E, Kostas S, Manzano D, Scheler U, Ferrer A, Tissier A, Makris AM, Kampranis SC, Kanellis AK (2015). Towards elucidating carnosic acid biosynthesis in *Lamiaceae*: functional characterization of the three first steps of the pathway in *Salvia fruticosa* and *Rosmarinus officinalis*. PLoS ONE.

[CR17] Cahoon EB, Ripp KG, Hall SE, McGonigle B (2002). Transgenic production of epoxy fatty acids by expression of a cytochrome P450 enzyme from *Euphorbia lagascae* seed. Plant Physiol.

[CR18] Cankar K, van Houwelingen A, Bosch D, Sonke T, Bouwmeester H, Beekwilder J (2011). A chicory cytochrome P450 mono-oxygenase CYP71AV8 for the oxidation of (+)-valencene. FEBS Lett.

[CR19] Carelli M, Biazzi E, Panara F, Tava A, Scaramelli L, Porceddu A, Graham N, Odoardi M, Piano E, Arcioni S, May S, Scotti C, Calderini O (2011). *Medicago truncatula* CYP716A12 is a multifunctional oxidase involved in the biosynthesis of hemolytic saponins. Plant Cell.

[CR20] Celedon JM, Chiang A, Yuen MM, Diaz-Chavez ML, Madilao LL, Finnegan PM, Barbour EL, Bohlmann J (2016). Heartwood-specific transcriptome and metabolite signatures of tropical sandalwood (*Santalum album*) reveal the final step of (*Z*)-santalol fragrance biosynthesis. Plant J.

[CR21] Chau M, Jennewein S, Walker K, Croteau R (2004). Taxol biosynthesis: molecular cloning and characterization of a cytochrome P450 taxoid 7β-hydroxylase. Chem Biol.

[CR22] Chen TC, Da Fonseca CO, Schönthal AH (2015). Preclinical development and clinical use of perillyl alcohol for chemoprevention and cancer therapy. Am. J. Cancer Res..

[CR23] Collu G, Unver N, Peltenburg-Looman AM, van der Heijden R, Verpoorte R, Memelink J (2001). Geraniol 10-hydroxylase, a cytochrome P450 enzyme involved in terpenoid indole alkaloid biosynthesis. FEBS Lett.

[CR24] de Carvalho CCCR, da Fonseca MMR (2006). Carvone: why and how should one bother to produce this terpene. Food Chem.

[CR25] Dejong JM, Liu Y, Bollon AP, Long RM, Jennewein S, Williams D, Croteau RB (2006). Genetic engineering of taxol biosynthetic genes in *Saccharomyces cerevisiae*. Biotechnol Bioeng.

[CR26] Diaz-Chavez ML, Moniodis J, Madilao LL, Jancsik S, Keeling CI, Barbour EL, Ghisalberti EL, Plummer JA, Jones CG, Bohlmann J (2013). Biosynthesis of Sandalwood Oil: *santalum album* CYP76F cytochromes P450 produce santalols and bergamotol. PLoS ONE.

[CR27] Dinda B, Debnath S, Banik R (2011). Naturally occurring iridoids and secoiridoids. An updated review, part 4. Chem Pharm Bull.

[CR28] Drew DP, Andersen TB, Sweetman C, Moller BL, Ford C, Simonsen HT (2016). Two key polymorphisms in a newly discovered allele of the *Vitis vinifera* TPS24 gene are responsible for the production of the rotundone precursor alpha-guaiene. J Exp Bot.

[CR29] Edgar S, Zhou K, Qiao K, King JR, Simpson JH, Stephanopoulos G (2016). Mechanistic insights into taxadiene epoxidation by taxadiene-5α-hydroxylase. ACS Chem Biol.

[CR30] Engels B, Dahm P, Jennewein S (2008). Metabolic engineering of taxadiene biosynthesis in yeast as a first step towards Taxol (Paclitaxel) production. Metab Eng.

[CR31] Facchini PJ, Chappell J (1992). Gene family for an elicitor-induced sesquiterpene cyclase in tobacco. Proc Natl Acad Sci USA.

[CR32] Forman V, Callari R, Folly C, Heider H, Hamberger B (2017) Production of putative diterpene carboxylic acid intermediates of triptolide in yeast. Molecules 22 10.3390/molecules22060981 PMC6152743 28608823

[CR33] Fukushima EO, Seki H, Ohyama K, Ono E, Umemoto N, Mizutani M, Saito K, Muranaka T (2011). CYP716A subfamily members are multifunctional oxidases in triterpenoid biosynthesis. Plant Cell Physiol.

[CR34] Funk C, Croteau R (1994). Diterpenoid resin acid biosynthesis in conifers: characterization of two cytochrome P450-dependent monooxygenases and an aldehyde dehydrogenase involved in abietic acid biosynthesis. Arch Biochem Biophys.

[CR35] Geisler K, Jensen NB, Yuen MM, Madilao L, Bohlmann J (2016). Modularity of conifer diterpene resin acid biosynthesis: P450 enzymes of different CYP720B clades use alternative substrates and converge on the same products. Plant Physiol.

[CR36] Geu-Flores F, Sherden NH, Courdavault V, Burlat V, Glenn WS, Wu C, Nims E, Cui Y, O’Connor SE (2012). An alternative route to cyclic terpenes by reductive cyclization in iridoid biosynthesis. Nature.

[CR37] Ginglinger JF, Boachon B, Hofer R, Paetz C, Kollner TG, Miesch L, Lugan R, Baltenweck R, Mutterer J, Ullmann P, Beran F, Claudel P, Verstappen F, Fischer MJC, Karst F, Bouwmeester H, Miesch M, Schneider B, Gershenzon J, Ehlting J, Werck-Reichhart D (2013). Gene coexpression analysis reveals complex metabolism of the monoterpene alcohol linalool in Arabidopsis flowers. Plant Cell.

[CR38] Greenhagen BT, Griggs P, Takahashi S, Ralston L, Chappell J (2003). Probing sesquiterpene hydroxylase activities in a coupled assay with terpene synthases. Arch Biochem Biophys.

[CR39] Guo J, Zhou YJ, Hillwig ML, Shen Y, Yang L, Wang Y, Zhang X, Liu W, Peters RJ, Chen X, Zhao ZK, Huang L (2013). CYP76AH1 catalyzes turnover of miltiradiene in tanshinones biosynthesis and enables heterologous production of ferruginol in yeasts. Proc Natl Acad Sci USA.

[CR40] Guo J, Ma X, Cai Y, Ma Y, Zhan Z, Zhou YJ, Liu W, Guan M, Yang J, Cui G, Kang L, Yang L, Shen Y, Tang J, Lin H, Ma X, Jin B, Liu Z, Peters RJ, Zhao ZK, Huang L (2016). Cytochrome P450 promiscuity leads to a bifurcating biosynthetic pathway for tanshinones. New Phytol.

[CR41] Hamberger B, Bak S (2013). Plant P450s as versatile drivers for evolution of species-specific chemical diversity. Phil. Trans. R. Soc. B.

[CR42] Hamberger B, Ohnishi T, Hamberger B, Seguin A, Bohlmann J (2011). Evolution of diterpene metabolism: Sitka spruce CYP720B4 catalyzes multiple oxidations in resin acid biosynthesis of conifer defense against insects. Plant Physiol.

[CR43] Hamberger, B., Møller, B. L., Pateraki, E., Andersen-Ranberg, J., Jensen, N. B., 2015. Biosynthesis of forskolin and related compounds. Google Patents; WO2015113569A1

[CR44] Hamberger, B., Andersen-Ranberg, J., Jensen, N. B., Pateraki, E., Møller, B. L., 2016. Biosynthesis of acetylated 13R-MO and related compounds. Google Patents; WO2016166243A1

[CR45] Haralampidis K, Trojanowska M, Osbourn AE (2002). Biosynthesis of triterpenoid saponins in plants. Adv Biochem Eng Biotechnol.

[CR46] Hefner J, Rubenstein SM, Ketchum REB, Gibson DM, Williams RM, Croteau R (1996). Cytochrome P450-catalyzed hydroxylation of taxa-4(5),11(12)-diene to taxa-4(20),11(12)-dien-5a-o1: the first oxygenation step in taxol biosynthesis. Chem Biol.

[CR47] Helliwell CA, Poole A, Peacock WJ, Dennis ES (1999). Arabidopsis *ent*-kaurene oxidase catalyzes three steps of gibberellin biosynthesis. Plant Physiol.

[CR48] Helliwell CA, Chandler PM, Poole A, Dennis ES, Peacock WJ (2001). The CYP88A cytochrome P450, *ent*-kaurenoic acid oxidase, catalyzes three steps of the gibberellin biosynthesis pathway. Proc Natl Acad Sci USA.

[CR49] Höfer R, Dong L, André F, Ginglinger J-F, Lugan R, Gavira C, Grec S, Lang G, Memelink J, Van Der Krol S, Bouwmeester H, Werck-Reichhart D (2013). Geraniol hydroxylase and hydroxygeraniol oxidase activities of the CYP76 family of cytochrome P450 enzymes and potential for engineering the early steps of the (seco)iridoid pathway. Metab Eng.

[CR50] Höfer R, Boachon B, Renault H, Gavira C, Miesch L, Iglesias J, Ginglinger JF, Allouche L, Miesch M, Grec S, Larbat R, Werck-Reichhart D (2014). Dual function of the cytochrome P450 CYP76 family from *Arabidopsis thaliana* in the metabolism of monoterpenols and phenylurea herbicides. Plant Physiol.

[CR51] Howat S, Park B, Oh IS, Jin YW, Lee EK, Loake GJ (2014). Paclitaxel: biosynthesis, production and future prospects. N. Biotechnol..

[CR52] Huang Q, Roessner CA, Croteau R, Scott AI (2001). Engineering *Escherichia coli* for the synthesis of taxadiene, a key intermediate in the biosynthesis of taxol. Bioorg Med Chem.

[CR53] Huang AC, Burrett S, Sefton MA, Taylor DK (2014). Production of the pepper aroma compound, (−)-rotundone, by aerial oxidation of alpha-guaiene. J Agric Food Chem.

[CR54] Huhman DV, Berhow MA, Sumner LW (2005). Quantification of saponins in aerial and subterranean tissues of *Medicago truncatula*. J Agric Food Chem.

[CR55] Ignea C, Athanasakoglou A, Ioannou E, Georgantea P, Trikka FA, Loupassaki S, Roussis V, Makris AM, Kampranis SC (2016). Carnosic acid biosynthesis elucidated by a synthetic biology platform. Proc Natl Acad Sci USA.

[CR56] Inoue Y, Sakai M, Yao Q, Tanimoto Y, Toshima H, Hasegawa M (2013). Identification of a novel casbane-type diterpene phytoalexin, *ent*-10-oxodepressin, from rice leaves. Biosci Biotechnol Biochem.

[CR57] Itkin M, Davidovich-Rikanati R, Cohen S, Portnoy V, Doron-Faigenboim A, Oren E, Freilich S, Tzuri G, Baranes N, Shen S, Petreikov M, Sertchook R, Ben-Dor S, Gottlieb H, Hernandez A, Nelson DR, Paris HS, Tadmor Y, Burger Y, Lewinsohn E, Katzir N, Schaffer A (2016). The biosynthetic pathway of the nonsugar, high-intensity sweetener mogroside V from *Siraitia grosvenorii*. Proc Natl Acad Sci USA.

[CR58] Jagalski V, Barker R, Topgaard D, Günther-Pomorski T, Hamberger B, Cárdenas M (2016). Biophysical study of resin acid effects on phospholipid membrane structure and properties. Biochim. Biophys. Acta - Biomembranes.

[CR59] Jennewein S, Croteau R (2001). Taxol: biosynthesis, molecular genetics, and biotechnological applications. Appl Microbiol Biotechnol.

[CR60] Jennewein S, Rithner CD, Williams RM, Croteau RB (2001). Taxol biosynthesis: taxane 13 alpha-hydroxylase is a cytochrome P450-dependent monooxygenase. Proc Natl Acad Sci USA.

[CR61] Jennewein S, Rithner CD, Williams RM, Croteau R (2003). Taxoid metabolism: taxoid 14β-hydroxylase is a cytochrome P450-dependent monooxygenase. Arch Biochem Biophys.

[CR62] Jennewein S, Long RM, Williams RM, Croteau R (2004). Cytochrome P450 taxadiene 5α-Hydroxylase, a mechanistically unusual monooxygenase catalyzing the first oxygenation step of taxol biosynthesis. Chem Biol.

[CR63] Jones CG, Moniodis J, Zulak KG, Scaffidi A, Plummer JA, Ghisalberti EL, Barbour EL, Bohlmann J (2011). Sandalwood fragrance biosynthesis involves sesquiterpene synthases of both the terpene synthase (TPS)-a and TPS-b subfamilies, including santalene synthases. J Biol Chem.

[CR64] Kaspera R, Croteau R (2006). Cytochrome P450 oxygenases of Taxol biosynthesis. Phytochem Rev.

[CR65] Kato H, Kodama O, Akatsuka T (1995). Characterization of an inducible P450 hydroxylase involved in the rice diterpene phytoalexin biosynthetic pathway. Arch Biochem Biophys.

[CR66] Keeling CI, Madilao LL, Zerbe P, Dullat HK, Bohlmann J (2011). The primary diterpene synthase products of *Picea abies* levopimaradiene/abietadiene synthase (*Pa*LAS) are epimers of a thermally unstable diterpenol. J Biol Chem.

[CR67] Khakimov B, Kuzina V, Erthmann PO, Fukushima EO, Augustin JM, Olsen CE, Scholtalbers J, Volpin H, Andersen SB, Hauser TP, Muranaka T, Bak S (2015). Identification and genome organization of saponin pathway genes from a wild crucifer, and their use for transient production of saponins in *Nicotiana benthamiana*. Plant J..

[CR68] King AJ, Brown GD, Gilday AD, Larson TR, Graham IA (2014). Production of bioactive diterpenoids in the euphorbiaceae depends on evolutionarily conserved gene clusters. Plant Cell.

[CR69] Kirby J, Keasling JD (2009). Biosynthesis of plant isoprenoids: perspectives for microbial engineering. Annu Rev Plant Biol.

[CR70] Kjonaas R, Croteau R (1983). Demonstration that limonene is the first cyclic intermediate in the biosynthesis of oxygenated *p*-menthane monoterpenes in *Mentha piperita* and other *Mentha* species. Arch Biochem Biophys.

[CR71] Komori A, Suzuki M, Seki H, Nishizawa T, Meyer JJ, Shimizu H, Yokoyama S, Muranaka T (2013). Comparative functional analysis of CYP71AV1 natural variants reveals an important residue for the successive oxidation of amorpha-4,11-diene. FEBS Lett.

[CR72] Kovacs K, Zhang L, Linforth RST, Whittaker B, Hayes CJ, Fray RG (2007). Redirection of carotenoid metabolism for the efficient production of taxadiene [taxa-4(5),11(12)-diene] in transgenic tomato fruit. Transgenic Res.

[CR73] Kries H, Caputi L, Stevenson CE, Kamileen MO, Sherden NH, Geu-Flores F, Lawson DM, O’Connor SE (2016). Structural determinants of reductive terpene cyclization in iridoid biosynthesis. Nat Chem Biol.

[CR74] LaFever RE, Vogel BS, Croteau R (1994). Diterpenoid resin acid biosynthesis in conifers: enzymatic cyclization of geranylgeranyl pyrophosphate to abietadiene, the precursor of abietic acid. Arch Biochem Biophys.

[CR75] Lange BM, Mahmoud SS, Wildung MR, Turner GW, Davis EM, Lange I, Baker RC, Boydston RA, Croteau RB (2011). Improving peppermint essential oil yield and composition by metabolic engineering. Proc Natl Acad Sci USA.

[CR76] Laursen T, Borch J, Knudsen C, Bavishi K, Torta F, Martens HJ, Silvestro D, Hatzakis NS, Wenk MR, Dafforn TR, Olsen CE, Motawia MS, Hamberger B, Moller BL, Bassard JE (2016). Characterization of a dynamic metabolon producing the defense compound dhurrin in sorghum. Science.

[CR77] Li Y, Pfeifer BA (2014). Heterologous production of plant-derived isoprenoid products in microbes and the application of metabolic engineering and synthetic biology. Curr Opin Plant Biol.

[CR78] Liu, Y., Hansen, J., Houghton-Larsen, J., Murali, M. P., Kumar, S., 2014. Methods and materials for biosynthesis of mogroside compounds (WO2014086842). Google Patents

[CR79] Luo D, Callari R, Hamberger B, Wubshet SG, Nielsen MT, Andersen-Ranberg J, Hallstrom BM, Cozzi F, Heider H, Lindberg Moller B, Staerk D, Hamberger B (2016). Oxidation and cyclization of casbene in the biosynthesis of Euphorbia factors from mature seeds of *Euphorbia lathyris* L. Proc Natl Acad Sci USA.

[CR80] Lupien S, Karp F, Wildung M, Croteau R (1999). Regiospecific cytochrome P450 limonene hydroxylases from mint (Mentha) species: cDNA isolation, characterization, and functional expression of (−)-4S-limonene-3-hydroxylase and (−)-4S-limonene-6-hydroxylase. Arch Biochem Biophys.

[CR81] Mafu S, Jia M, Zi J, Morrone D, Wu Y, Xu M, Hillwig ML, Peters RJ (2016). Probing the promiscuity of *ent*-kaurene oxidases via combinatorial biosynthesis. Proc Natl Acad Sci USA.

[CR82] Mahmoud SS, Croteau RB (2001). Metabolic engineering of essential oil yield and composition in mint by altering expression of deoxyxylulose phosphate reductoisomerase and menthofuran synthase. Proc Natl Acad Sci USA.

[CR83] Martin DM, Aubourg S, Schouwey MB, Daviet L, Schalk M, Toub O, Lund ST, Bohlmann J (2010). Functional annotation, genome organization and phylogeny of the grapevine (*Vitis vinifera*) terpene synthase gene family based on genome assembly, FLcDNA cloning, and enzyme assays. BMC Plant Biol.

[CR84] Matsuba Y, Nguyen TT, Wiegert K, Falara V, Gonzales-Vigil E, Leong B, Schafer P, Kudrna D, Wing RA, Bolger AM, Usadel B, Tissier A, Fernie AR, Barry CS, Pichersky E (2013). Evolution of a complex locus for terpene biosynthesis in solanum. Plant Cell.

[CR85] Mau CJ, Karp F, Ito M, Honda G, Croteau RB (2010). A candidate cDNA clone for (−)-limonene-7-hydroxylase from *Perilla frutescens*. Phytochemistry.

[CR86] Mizutani M, Sato F (2011). Unusual P450 reactions in plant secondary metabolism. Arch Biochem Biophys.

[CR87] Moses T, Pollier J, Shen Q, Soetaert S, Reed J, Erffelinck ML, Van Nieuwerburgh FC, Vanden Bossche R, Osbourn A, Thevelein JM, Deforce D, Tang K, Goossens A (2015). OSC2 and CYP716A14v2 catalyze the biosynthesis of triterpenoids for the cuticle of aerial organs of *Artemisia annua*. Plant Cell.

[CR88] Naoumkina MA, Modolo LV, Huhman DV, Urbanczyk-Wochniak E, Tang Y, Sumner LW, Dixon RA (2010). Genomic and coexpression analyses predict multiple genes involved in triterpene saponin biosynthesis in *Medicago truncatula*. Plant Cell.

[CR89] Nelson D, Werck-Reichhart D (2011). A P450-centric view of plant evolution. Plant J..

[CR90] Nielsen AZ, Mellor SB, Vavitsas K, Wlodarczyk AJ, Gnanasekaran T, de Perestrello Ramos H, Jesus M, King BC, Bakowski K, Jensen PE (2016). Extending the biosynthetic repertoires of cyanobacteria and chloroplasts. Plant J..

[CR91] Nomura T, Magome H, Hanada A, Takeda-Kamiya N, Mander LN, Kamiya Y, Yamaguchi S (2013). Functional analysis of *Arabidopsis* CYP714A1 and CYP714A2 reveals that they are distinct gibberellin modification enzymes. Plant Cell Physiol.

[CR92] Oye KA, Lawson JC, Bubela T (2015). Drugs: regulate ‘home-brew’ opiates. Nature.

[CR93] Parage C, Foureau E, Kellner F, Burlat V, Mahroug S, Lanoue A, Duge de Bernonville T, Londono MA, Carqueijeiro I, Oudin A, Besseau S, Papon N, Glevarec G, Atehortua L, Giglioli-Guivarc’h N, St-Pierre B, Clastre M, O’Connor SE, Courdavault V (2016). Class II cytochrome P450 reductase governs the biosynthesis of alkaloids. Plant Physiol.

[CR94] Pateraki I, Andersen-Ranberg J, Hamberger B, Heskes AM, Martens HJ, Zerbe P, Bach SS, Moller BL, Bohlmann J, Hamberger B (2014). Manoyl oxide (13R), the biosynthetic precursor of forskolin, is synthesized in specialized root cork cells in Coleus forskohlii. Plant Physiol.

[CR95] Pateraki I, Heskes AM, Hamberger B (2015). Cytochromes P450 for terpene functionalisation and metabolic engineering. Adv Biochem Eng Biotechnol.

[CR96] Pateraki, I., Andersen-Ranberg, J., Jensen, N. B., Wubshet, S. G., Heskes, A. M., Forman, V., Hallstrom, B., Hamberger, B., Motawia, M. S., Olsen, C. E., Staerk, D., Hansen, J., Moller, B. L., Hamberger, B., 2017. Total biosynthesis of the cyclic AMP booster forskolin from *Coleus forskohlii*. Elife 6 10.7554/eLife.23001 PMC5388535 28290983

[CR97] Peplow M (2016). Synthetic biology’s first malaria drug meets market resistance. Nature.

[CR98] Ralston L, Kwon ST, Schoenbeck M, Ralston J, Schenk DJ, Coates RM, Chappell J (2001). Cloning, heterologous expression, and functional characterization of 5-*epi*-aristolochene-1,3-dihydroxylase from tobacco (*Nicotiana tabacum*). Arch Biochem Biophys.

[CR99] Renault H, Bassard J-E, Hamberger B, Werck-Reichhart D (2014). Cytochrome P450-mediated metabolic engineering: current progress and future challenges. Curr Opin Plant Biol.

[CR100] Ringer KL, McConkey ME, Davis EM, Rushing GW, Croteau R (2003). Monoterpene double-bond reductases of the (−)-menthol biosynthetic pathway: isolation and characterization of cDNAs encoding (−)-isopiperitenone reductase and (+)-pulegone reductase of peppermint. Arch Biochem Biophys.

[CR101] Ringer KL, Davis EM, Croteau R (2005). Monoterpene metabolism. Cloning, expression, and characterization of (−)-isopiperitenol/(−)-carveol dehydrogenase of peppermint and spearmint. Plant Physiol.

[CR102] Rios-Estepa R, Turner GW, Lee JM, Croteau RB, Lange BM (2008). A systems biology approach identifies the biochemical mechanisms regulating monoterpenoid essential oil composition in peppermint. Proc Natl Acad Sci USA.

[CR103] Ro DK, Arimura G, Lau SY, Piers E, Bohlmann J (2005). Loblolly pine abietadienol/abietadienal oxidase *Pt*AO (CYP720B1) is a multifunctional, multisubstrate cytochrome P450 monooxygenase. Proc Natl Acad Sci USA.

[CR104] Ro DK, Paradise EM, Ouellet M, Fisher KJ, Newman KL, Ndungu JM, Ho KA, Eachus RA, Ham TS, Kirby J, Chang MCY, Withers ST, Shiba Y, Sarpong R, Keasling JD (2006). Production of the antimalarial drug precursor artemisinic acid in engineered yeast. Nature.

[CR105] Roh HS, Lim EG, Kim J, Park CG (2011). Acaricidal and oviposition deterring effects of santalol identified in sandalwood oil against two-spotted spider mite, *Tetranychus urticae* Koch (Acari: Tetranychidae). J Pest Sci.

[CR106] Rontein D, Onillon S, Herbette G, Lesot A, Werck-Reichhart D, Sallaud C, Tissier A (2008). CYP725A4 from yew catalyzes complex structural rearrangement of taxa-4(5),11(12)-diene into the cyclic ether 5(12)-oxa-3(11)-cyclotaxane. J Biol Chem.

[CR107] Schalk M, Croteau R (2000). A single amino acid substitution (F363I) converts the regiochemistry of the spearmint (−)-limonene hydroxylase from a C6- to a C3-hydroxylase. Proc Natl Acad Sci USA.

[CR108] Scheler U, Brandt W, Porzel A, Rothe K, Manzano D, Bozic D, Papaefthimiou D, Balcke GU, Henning A, Lohse S, Marillonnet S, Kanellis AK, Ferrer A, Tissier A (2016). Elucidation of the biosynthesis of carnosic acid and its reconstitution in yeast. Nat. Commun..

[CR109] Schilmiller AL, Schauvinhold I, Larson M, Xu R, Charbonneau AL, Schmidt A, Wilkerson C, Last RL, Pichersky E (2009). Monoterpenes in the glandular trichomes of tomato are synthesized from a neryl diphosphate precursor rather than geranyl diphosphate. Proc Natl Acad Sci USA.

[CR110] Schmelz EA, Huffaker A, Sims JW, Christensen SA, Lu X, Okada K, Peters RJ (2014). Biosynthesis, elicitation and roles of monocot terpenoid phytoalexins. Plant J..

[CR111] Schoendorf A, Rithner CD, Williams RM, Croteau RB (2001). Molecular cloning of a cytochrome P450 taxane 10 beta-hydroxylase cDNA from *Taxus* and functional expression in yeast. Proc Natl Acad Sci USA.

[CR112] Seki H, Ohyama K, Fau-Sawai S, Sawai S, Mizutani M, Ohnishi T, Sudo H, Akashi T, Aoki T, Saito K, Muranaka T (2008). Licorice beta-amyrin 11-oxidase, a cytochrome P450 with a key role in the biosynthesis of the triterpene sweetener glycyrrhizin. Proc Natl Acad Sci USA.

[CR113] Seki H, Tamura K, Muranaka T (2015). P450s and UGTs: key players in the structural diversity of triterpenoid saponins. Plant Cell Physiol.

[CR114] Shang Y, Ma Y, Zhou Y, Zhang H, Duan L, Chen H, Zeng J, Zhou Q, Wang S, Gu W, Liu M, Ren J, Gu X, Zhang S, Wang Y, Yasukawa K, Bouwmeester HJ, Qi X, Zhang Z, Lucas WJ, Huang S (2014). Plant science. Biosynthesis, regulation, and domestication of bitterness in cucumber. Science.

[CR115] Shibuya M, Hoshino M, Katsube Y, Hayashi H, Kushiro T, Ebizuka Y (2006). Identification of β-amyrin and sophoradiol 24-hydroxylase by expressed sequence tag mining and functional expression assay. FEBS J.

[CR116] Sparg SG, Light ME, van Staden J (2004). Biological activities and distribution of plant saponins. J Ethnopharmacol.

[CR117] Suzuki H, Achnine L, Xu R, Matsuda SPT, Dixon RA (2002). A genomics approach to the early stages of triterpene saponin biosynthesis in *Medicago truncatula*. Plant J..

[CR118] Takahashi S, Zhao Y, O’Maille PE, Greenhagen BT, Noel JP, Coates RM, Chappell J (2005). Kinetic and molecular analysis of 5-epiaristolochene 1,3-dihydroxylase, a cytochrome P450 enzyme catalyzing successive hydroxylations of sesquiterpenes. J Biol Chem.

[CR119] Takahashi S, Yeo YS, Zhao Y, O’Maille PE, Greenhagen BT, Noel JP, Coates RM, Chappell J (2007). Functional characterization of premnaspirodiene oxygenase, a cytochrome P450 catalyzing regio- and stereo-specific hydroxylations of diverse sesquiterpene substrates. J Biol Chem.

[CR120] Takase H, Sasaki K, Shinmori H, Shinohara A, Mochizuki C, Kobayashi H, Ikoma G, Saito H, Matsuo H, Suzuki S, Takata R (2016). Cytochrome P450 CYP71BE5 in grapevine (*Vitis vinifera*) catalyzes the formation of the spicy aroma compound (−)-rotundone. J Exp Bot.

[CR121] Tamura K, Seki H, Suzuki H, Kojoma M, Saito K, Muranaka T (2017). CYP716A179 functions as a triterpene C-28 oxidase in tissue-cultured stolons of *Glycyrrhiza uralensis*. Plant Cell Rep.

[CR122] Tava A, Avato P (2006). Chemical and biological activity of triterpene saponins from *Medicago* species. Nat Prod Commun.

[CR123] Tundis R, Loizzo MR, Menichini F, Statti GA, Menichini F (2008). Biological and pharmacological activities of iridoids: recent developments. Mini Rev Med Chem.

[CR124] Viljoen A, Mncwangi N, Vermaak I (2012). Anti-inflammatory iridoids of botanical origin. Curr Med Chem.

[CR125] Walker K, Croteau R (2001). Taxol biosynthetic genes. Phytochemistry.

[CR126] Wang J, Li S, Xiong Z, Wang Y (2016). Pathway mining-based integration of critical enzyme parts for de novo biosynthesis of steviolglycosides sweetener in *Escherichia coli*. Cell Res.

[CR127] Wani MC, Taylor HL, Wall ME, Coggon P, McPhail AT (1971). Plant antitumor agents. VI. The isolation and structure of taxol, a novel antileukemic and antitumor agent from *Taxus brevifolia*. J Am Chem Soc.

[CR128] Wildung MR, Croteau R (1996). A cDNA clone for taxadiene synthase, the diterpene cyclase that catalyzes the committed step of taxol biosynthesis. J Biol Chem.

[CR129] Wood C, Siebert TE, Parker M, Capone DL, Elsey GM, Pollnitz AP, Eggers M, Meier M, Vossing T, Widder S, Krammer G, Sefton MA, Herderich MJ (2008). From wine to pepper: rotundone, an obscure sesquiterpene, is a potent spicy aroma compound. J Agric Food Chem.

[CR130] Yasumoto S, Fukushima EO, Seki H, Muranaka T (2016). Novel triterpene oxidizing activity of *Arabidopsis thaliana* CYP716A subfamily enzymes. FEBS Lett.

[CR131] Zerbe P, Hamberger B, Yuen MM, Chiang A, Sandhu HK, Madilao LL, Nguyen A, Hamberger B, Bach SS, Bohlmann J (2013). Gene discovery of modular diterpene metabolism in nonmodel systems. Plant Physiol.

[CR132] Zhang J, Dai L, Yang J, Liu C, Men Y, Zeng Y, Cai Y, Zhu Y, Sun Y (2016). Oxidation of cucurbitadienol catalyzed by CYP87D18 in the biosynthesis of mogrosides from *Siraitia grosvenorii*. Plant Cell Physiol.

[CR133] Zhou K, Qiao K, Edgar S, Stephanopoulos G (2015). Distributing a metabolic pathway among a microbial consortium enhances production of natural products. Nat Biotechnol.

[CR134] Zhou Y, Ma Y, Zeng J, Duan L, Xue X, Wang H, Lin T, Liu Z, Zeng K, Zhong Y, Zhang S, Hu Q, Liu M, Zhang H, Reed J, Moses T, Liu X, Huang P, Qing Z, Liu X, Tu P, Kuang H, Zhang Z, Osbourn A, Ro DK, Shang Y, Huang S (2016). Convergence and divergence of bitterness biosynthesis and regulation in Cucurbitaceae. Nat. Plants.

[CR135] Zi J, Peters RJ (2013). Characterization of CYP76AH4 clarifies phenolic diterpenoid biosynthesis in the Lamiaceae. Org Biomol Chem.

[CR136] Zi J, Mafu S, Peters RJ (2014). To gibberellins and beyond! Surveying the evolution of (di)terpenoid metabolism. Annu Rev Plant Biol.

